# Preparative isolation and preliminary characterization of LHCII trimers and PSII monomers from *Posidonia oceanica* L.

**DOI:** 10.1007/s11120-026-01196-3

**Published:** 2026-01-29

**Authors:** Stefano Francesco Farci, Luca Iesu, Domenica Farci, Dario Piano

**Affiliations:** 1 Department of Life and Environmental Sciences, University of Cagliari, Cagliari, Italy; 2 Université Paris-Saclay, Univ Evry, CY Cergy Paris Université, CNRS, LAMBE, Cergy, 95000 France; 3 Department of Plant Physiology, Warsaw University of Life Sciences SGGW, Warsaw, Poland

**Keywords:** Light harvesting complex, Photosystem II, *Posidonia oceanica* L, Polyphenols extraction, Gene annotation, Mass spectrometry, Anion exchange chromatography

## Abstract

*Posidonia oceanica* L. is a vascular marine plant that represents a relevant case study in plant physiology due to its phylogenetic position, ecological adaptations, and, in particular, the specialization of its photosynthetic apparatus. Here, we present a procedure for fractional thylakoid solubilization, in which a crude leaf extract obtained by blending is preliminarily depleted of the polyphenolic fraction and subsequently subjected to stepwise solubilization. The resulting thylakoid extracts, processed through a chromatographic workflow, enable the preparative isolation of trimeric Light-Harvesting Complex II and monomeric Photosystem II core complex. Protein identification was supported by mass spectrometry, with peptide homology analysis used for the assignment due to the absence of a curated proteome for *P. oceanica*. The resulting fractions enable downstream characterization, establishing the first chromatography-based preparative workflow for functional and structural investigations of the photosynthetic apparatus of this ecologically unique species.

**Supplementary Information:**

The online version contains supplementary material available at 10.1007/s11120-026-01196-3.

## Introduction

The seagrass phanerogam *Posidonia oceanica* L. is an endemic vascular plant of the Mediterranean Sea (Gobert 2006). This species forms extensive meadows that act as ecological scaffolds, supporting complex networks of biodiversity and giving rise to distinctive associated ecosystems (Abadie et al. 2018; García-Gómez et al. 2022). *P. oceanica* meadows are biodiversity hotspots, hosting approximately 20–25% of all marine Mediterranean species (Poli et al. 2020). As a key structural component of the marine environment, it provides organisms with both a major source of primary production and protective shelter (Abadie et al. 2018; Rigo et al. 2021; García-Gómez et al. 2022).

*P. oceanica* meadows typically extend from a few meters below the surface down to 30–40 m, with maximum depths of 45–50 m in exceptionally clear waters, reflecting both the wide ecological distribution of the species and its strong dependence on light availability (Duarte 1991). In this context, *P. oceanica* exhibits a broad adaptive range and marked photophysiological plasticity, enabling its photosynthetic apparatus to optimize performance across varying bathymetric gradients and light regimes (Dattolo et al. 2013; Procaccini et al. 2017; Madonia et al. 2021). Light composition is indeed strongly influenced by water turbidity and diffusion processes across the bathymetric profile, leading to a gradual reduction in its intensity with depth, as well as a shift in spectral quality, with progressive depletion of the red wavelengths and an increasing dominance of the blue fraction (Kirk 2011). This photophysiological plasticity has profound consequences, resulting in functional and compositional variability of the photosynthetic apparatus among individuals in meadows, depending on their bathymetric position. Moreover, it underpins the ecological success of *P. oceanica*, allowing meadows to maintain high primary productivity, habitat complexity, and energy flow across variable depths and light conditions (Koopmans et al. 2020). Given these aspects, important differences in the organization of the photosynthetic complexes in this species are to be expected. The energy partition in the photosynthetic apparatus of this species may differ not only in its external antenna distribution and related state transition processes, as observed in model organisms (Wood and Johnson 2020; Cutolo et al. 2023), but also in major structural features of the individual complexes, including cofactor orientation and their potentially variable qualitative and quantitative composition.

Despite this species has been extensively investigated on its ecological role in the last decades, a comprehensive structural and functional characterization of its photosynthetic apparatus is still missing. Previous ecophysiological studies provided partial insights into the general properties and photosynthetic performance of *P. oceanica*, but detailed structural and functional analyses remain scarce (Madonia et al. 2021). One of the main limiting factors is its coriaceous leaf, which hinder the isolation of thylakoids and their components. In addition, the abundant content of polyphenols and other secondary metabolites further interferes with isolation and characterization (Ozdal et al. 2013; Adamczyk et al. 2017; Seker et al. 2019; Liu et al. 2019; Bag et al. 2021), particularly in spectroscopical studies (Sęczyk et al. 2019; Benito-González et al. 2019; Astudillo-Pascual et al. 2021).

Although draft genomic assemblies and transcriptomic datasets exist for *P. oceanica* (Dattolo et al. 2013; Ma et al. 2024), a fully reference genome has not yet been resolved *(Posidonia oceanica* Genome Project - IMEDEA*).* This limitation is amplified at the proteomic level, where only 199 protein-level entries are currently available in UniProt. Consequently, peptide identification must be often inferred by homology to species with well-annotated proteomes, introducing a substantial degree of uncertainty in sequence assignment. This represents a potentially critical bottleneck for high-resolution structural biology on this species.

To date, only two experimental attempts have been published on the isolation of intact chloroplasts or thylakoids in this species (Piro et al. 2015; Charras et al. 2024), which represent valuable and relevant references, whereas several procedures for thylakoid isolation from conifers, also characterized by polyphenolic and coriaceous leaf, represent important methodological starting points (Bag et al. 2021). Here, after depletion of polyphenolic components, thylakoids from *P. oceanica* are subject to two subsequent solubilizations (Table 1). The solubilized fractions are then processed through a preparative chromatographic workflow, yielding samples enriched in Light-Harvesting Complex II (LHCII) from the first solubilization pool and samples enriched in Photosystem II (PSII) from the second solubilization. The procedure is preparative and scalable, overcoming the intrinsic limitations of previously reported non-preparative sucrose-gradient approaches (Charras-Ferroussier et al. 2025). The two chromatographic fractions obtained by these procedures were analyzed by absorption spectroscopy, electrophoresis, and mass spectrometry, allowing the identification of the complexes and their oligomeric state as LHCII trimers and monomeric PSII, respectively. The procedure provides sufficient material for downstream structural and functional analyses of LHCII and PSII, two complexes expected to significantly contribute to the photophysiological plasticity of *P. oceanica*.

**Table 1:** Workflow summarizing the Stepwise extraction, washing, and detergent solubilization of P. *oceanica* leaf material. The table indicates starting and resulting materials, intermediate compositions, removed fractions, process steps, and processing agents used to progressively remove polyphenols and fractionate thylakoid membranes

Starting material	Resulting material	Resulting material composition		Process Step	Processing agent	Downstream assay
		Process intermediate	Removed fraction (to waste or to analyze)			
Leaf	Crude extract (I) (solid fraction)	Leaf’s debris + cell walls + membranes	grinding buffer + cytosol (discharged)	Blending and separation	Waring blender and Centrifuge	None
Crude extract (I)	Crude extract (II)	as above but with > 50% polyphenols removed*	grinding buffer + PEG4000 50% + Polyphenols (discharged)	Extraction and washing	PEG4000 50% and Centrifuge	None
Crude extract (II)	Crude extract (II)	as above but with > 75% polyphenols removed*	grinding buffer + Polyphenols (discharged)	Washing	Centrifuge	Absorption spectroscopy
		as above but with > 90% polyphenols removed*	grinding buffer + Polyphenols (discharged)			
		as above but polyphenols free	grinding buffer + Polyphenols (discharged)			
Crude extract (II)	Crude extract (III)	as above but depleted of a first thylakoid fraction	Fraction A of solubilized thylakoids (analyzed)	Solubilization A and separation	β -Dodecylmaltoside 25 mM and centrifuge	Absorption spectroscopy; Denaturing PAGE; Ion Exchange Chromatography followed by Absorption spectroscopy, BN-PAGE, and MS
Crude extract (III)	Fraction recalcitrant to solubilization	as above but depleted of a second thylakoid fraction	Fraction B of solubilized thylakoids (analyzed)	Solubilization B and separation	β -Dodecylmaltoside 25 mM and centrifuge	Absorption spectroscopy; Denaturing PAGE; Ion Exchange Chromatography followed by Absorption spectroscopy, BN-PAGE, and MS

**Fig. 1:**
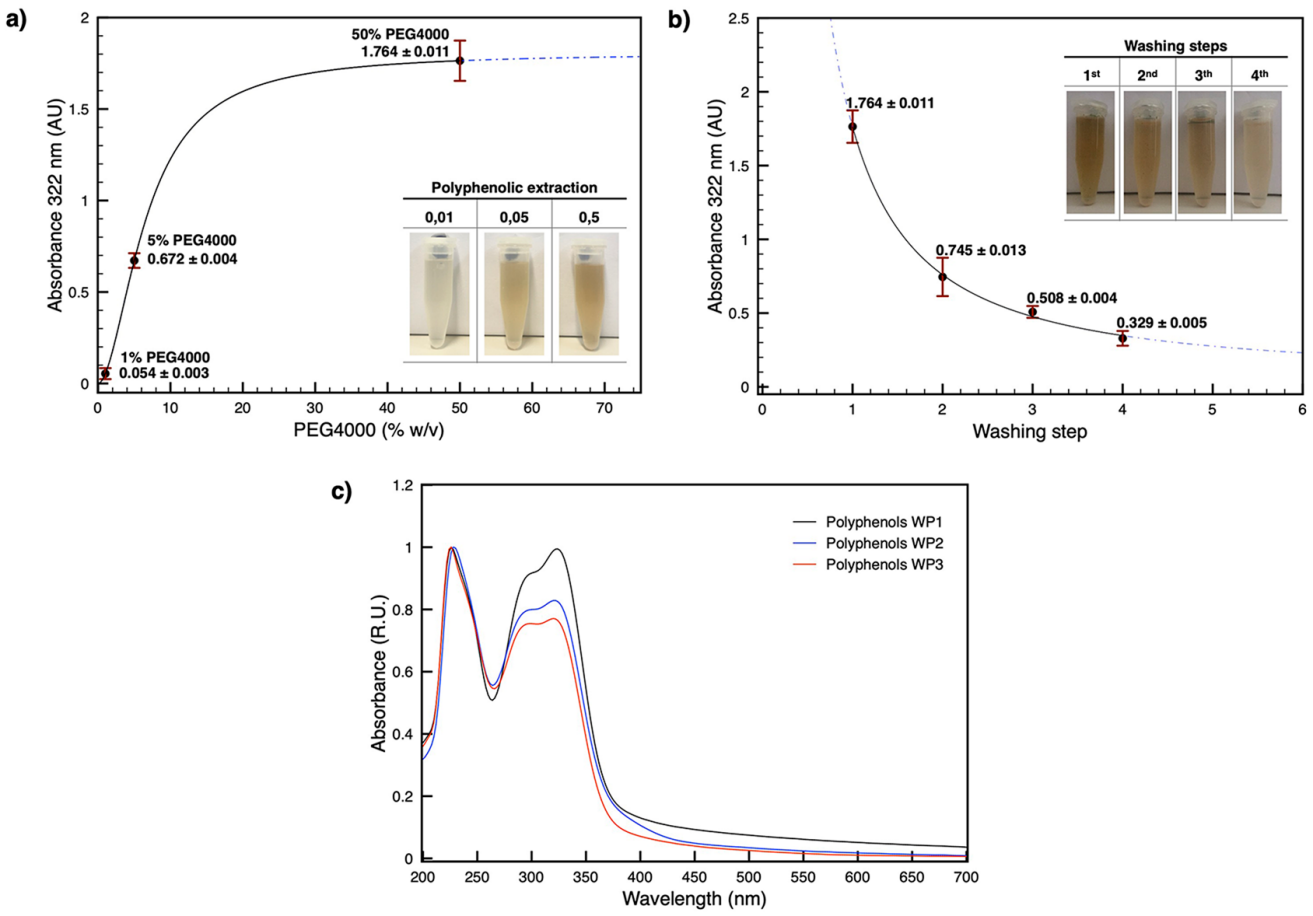
Efficiency of polyphenols removal as a function of PEG 4000 concentration and related washing steps. (**a**) Polyphenols extraction with PEG4000 was tested at 1%, 5%, and 50% concentration. Extraction efficiency was assessed by measuring the absorbance at 322 nm for each condition, showing increased polyphenols extraction from low to high PEG concentrations. Data points correspond to the polyphenol content (mean ± SEM) at 1%, 5%, and 50% PEG4000. The inset shows representative extractions at 1%, 5%, and 50% PEG4000, with color intensity increasing together with the PEG concentration. The dot-dashed line indicates the resulting curve fitting. (**b**) Effect of successive washing steps following treatment with 50% PEG 4000, assessed by absorbance measurements at 322 nm. A progressive decrease in absorbance is observed from the first to the fourth washing step, indicating the gradual and exhaustive removal of polyphenols. The inset shows the corresponding washing fractions. The dot-dashed line indicates the resulting curve fitting. (**c**) UV–Vis absorption spectra (200–700 nm) of the three sequential washing phases (WP1, WP2, and WP3) following PEG4000 extraction. All spectra show peaks at 226 nm and 322 nm, with a shoulder at 296 nm. Quantification in (**a**) and (**b**) is based on the absorbance at 322 nm

*Linear approximation of the absorbances values measured during the washing steps upon polyphenols extraction (Fig. 1)

Given the absence of a curated proteome database, peptide identification was mostly carried out by homology against proteomic datasets from well-annotated species. Therefore, the procedure is not only a preparative, scalable method for the thylakoid extraction and subsequent isolation of photosynthetic complexes from *P. oceanica* but also provides experimental evidence contributing to the inference of the most plausible protein sequences for both LHCII and PSII subunits from the available genomic and transcriptomic resources of *P. oceanica*. This preliminary study represents a contribution to the field toward a detailed structural and functional characterization of the photosynthetic complexes in this species, a key player in the Mediterranean marine ecosystems.

## Materials and methods

### Collection of plant material

Leaf of *P. oceanica* were collected at night as accidental bycatch from fishing nets at the same location (GPS coordinates 39.181947, 9.152182), at a depth of approximately 15 m with a surface temperature ranging between 19 and 22 °C. Sampling was conducted between July and September (2022–2025), and the material was transported to the laboratory for processing within 2–3 h. During transport, the leaf material was kept in seawater at temperatures not exceeding 16 °C and in the dark.

### Leaf preparation and thylakoids solubilization

Upon arrival in the laboratory, all the procedures were performed under dim green light. The leaf material was washed twice with MilliQ water, and healthy portions of young leaf, free of necrotic or chlorotic areas, were selected. The selected leaf fragments were cut into small pieces, 1–2 cm in length, and washed once more with MilliQ water. The washed fragments were then blended in cold (4 °C) Grinding Buffer (GB: 50 mM MES, pH 6.5; 10 mM MgCl_2_·6H_2_O; 10 mM CaCl_2_·2H_2_O) at a leaf-to-buffer ratio of 1:2 (w/v) by three consecutive steps of 1 min each. The resulting suspension of leaf fragments was centrifuged at 5000 × g for 10 min at 4 °C, and the pellet was resuspended in twice the initial volume of GB (1:4 w/v ratio respect to the initial mass of blended leaf) supplemented with 50% PEG 4000 (trials at 1% and 5% PEG 4000 were also done) to facilitate extraction of the polyphenolic fraction. After 30 min of mixing, the sample was centrifuged at 5000 × g for 10 min at 4 °C and the resulting pellet was resuspended in GB; this resuspension and centrifugation step was repeated up to three more times in GB, until the extract appeared clear or pale brown/yellow instead of dark brown. The final pellet was resuspended in GB at a ratio of approximately 1:4 (w/v) and solubilized in β-DDM to a final concentration of 2% in the dark at 4 °C for 30 min under gentle mixing on a rocking mixer. Following solubilization, the sample was centrifuged at 28,000 × g for 10 min at 4 °C, and the resulting supernatant, referred to as solubilization A, was collected. The pellet was then processed in the same manner a second time, eventually obtaining a solubilization B.

### Denaturing and native electrophoresis

Denaturing Tris-Tricine Sodium Dodecyl Sulphate-polyacrylamide gel electrophoresis (SDS-PAGE) was performed in the presence of 6 M Urea, using 10% (w/v) separating and 4% (w/v) stacking gels (Fantuzzi et al. 2023). Similarly, Lithium Dodecyl Sulphate-PAGE (LiDS-PAGE) was performed in presence of 6 M and 8 M Urea, using 10% (w/v) separating and 4% (w/v) stacking gels. Prior to loading, samples were subjected to standard denaturation by adding ROTIload 1 (Roth) and boiling for 10 min. Electrophoretic separation was carried out at 80 V for 3 h. After separation, gels were removed, rinsed with distilled water, and stained with Coomassie Brilliant Blue (CBB) G-250.

Blue Native-Polyacrylamide Gel Electrophoresis (BN-PAGE) was conducted using 3–12% (w/v) continuous gradient gels, following established protocols (Farci et al. 2017). Samples were treated in a ration 1:10 with a native CBB loading buffer (5% (v/v) Serva Blue G, 750 mM aminocaproic acid, 35% (w/v) sucrose). Electrophoretic separation was performed at 60 V for 12 h at 4 °C.

### Anion-exchange chromatography

For anion-exchange chromatography, a hand-packed column with Q sepharose High Performance resin (Cytiva, Marlborough, MA, USA and Uppsala, Sweden) was equilibrated in Washing Buffer (50 mM MES pH 6.5, 10 mM CaCl_2_·2H_2_O, 10 mM MgCl_2_·6H_2_O) supplemented with 0.03% (w/v) β-DDM at a flow rate of 0.5 mL/min. After injection, washing step was performed at a flow rate of 0.5 mL/min for 5 column volumes until the absorbance was stable to zero. Finally, the bound components were eluted in a 4 column volumes gradient of 0–100% Elution Buffer (50 mM MES pH 6.5, 10 mM CaCl_2_·2H_2_O, 10 mM MgCl_2_·6H_2_O, 2.5 M NaCl) supplemented with 0.03% (w/v) β-DDM at a flow rate of 0.5 mL/min.

### *In-gel* mass spectrometry

The bands from native electrophoresis were excised and subjected to LC-MS/MS analysis. Excised bands were destained, washed and subjected to a mixing step of protein reduction (10 mM DTT in 25 mM NaHCO_3_, 45 min, 56 °C, 750 rpm) and alkylation (55 mM iodoacetamide in 25 mM NaHCO_3_; 30 min, laboratory temperature, 750 rpm). After further washing by 50% acetonitrile/NaHCO_3_ and pure acetonitrile, the gel pieces were incubated with 125 ng trypsin (SOLu-Trypsin; Sigma Aldrich) in 50 mM NaHCO_3_. The digestion was performed for 2 h at 40 °C on a Thermomixer (750 rpm; Eppendorf). Tryptic peptides were extracted into LC-MS vials by 2.5% formic acid in 50% acetonitrile with addition of polyethylene glycol 20,000 (PEG 20000; final concentration 0.001%) and concentrated in a SpeedVac concentrator (Thermo Fisher Scientific). LC-MS/MS analyses of the peptide mixtures were conducted using an UltiMate 3000 RSLCnano system (Thermo Fisher Scientific) connected to a timsTOF Pro spectrometer (Bruker). After de-salting on a µPrecolumn (300 μm ID, 5 mm long, Thermo Fisher Scientific) the peptides were separated using an analytical column Aurora C18 (25 cm long, 75 μm inner diameter, 1.7 μm particles) under flow rate of 150 nL/min at 50 °C. Mobile phase A consisted of 0.1% formic acid in water, and mobile phase B was composed of 0.1% formic acid in 80% acetonitrile. After 4 min in 3% mobile phase B its content was linearly increased to 42% during the next 90 min, while after the separation, a wash step with 80% mobile phase B was performed. The analytical column was maintained inside a Column Toaster (Bruker), and the emitter side was connected to the CaptiveSpray ion source (Bruker). MS/MS data were acquired using a data-dependent acquisition with parallel accumulation–serial fragmentation (DDA-PASEF) method. Mascot MS/MS ion searches (Matrix Science, London, UK; version 3.1) were conducted against a database of protein sequences from *Posidonia oceanica*, which was downloaded from www.ncbi.nlm.nih.gov/protein/. Additionally, searches were performed against SwissProt database without taxonomy restrictions. The cRAP database (downloaded from http://www.thegpm.org/crap/) was searched in parallel to eliminate contaminant spectra. The mass tolerance for precursor ions and MS/MS fragment ions was set to 10 ppm and 0.1 Da, respectively, with the option to include one ^13^C atom in the parent ion. Oxidation of methionine was specified as a variable modification, while carbamidomethylation of cysteine was designated as a fixed modification. The estimate representativity of each entry was derived from an index calculated by dividing the MASCOT score by the protein mass (kDa), followed by normalization relative to the highest ranked entry corresponding to a subunit of the putative complex in the native gel band, defined based on its apparent mass (PSII subunits for the ~ 200 kDa bands A1 and B1; LHCII subunits for the ~ 70 kDa bands A2 and B2), and which was set to 1. Proteins were then ranked according to this normalized index to highlight their qualitative abundance and, consequently, their representativeness within each BN-PAGE band.

### Absorption spectroscopy

Measurements were done using a Vis ONDA Touch V-11 Scan spectrophotometer, set with a 1 nm bandpass on a quartz cuvette with a optical path length of 1 cm. Absorption spectra of solubilized thylakoid membranes and polyphenolic extracts were recorded in the range 350–750 nm and 200–700 nm respectively. For polyphenols, the absorption maximum at 322 nm, experimentally identified (Fig. 1c), was used to quantify the efficiency of the extraction with PEG4000 1%, 5% and 50% in GB. The efficiency of the four washing steps following the 50% extraction (selected as the most efficient among the three previously tested), was also evaluated using the absorption at 322 nm. All reported values represent the mean of three technical replicates collected from the same experiment and condition. The means were then used to calculate the Standard Error of the Mean (SEM) across at least three independent experiments performed for different thylakoid preparations.

## Results

### The use of PEG 4000 enables polyphenolic removal from blended leaf

As a first step, the leaf was blended and the polyphenolic secondary metabolites were removed to both facilitate solubilization and prevent interference with the subsequent steps. To achieve this, the blended leaf material was subjected to centrifugation, and the resulting pellet was washed by incubation for 30 min under continuous mixing in a buffer containing PEG 4000. Specifically, the grinding buffer used for blending the leaf was supplemented with PEG 4000 at concentrations of 1%, 5%, and 50%. After incubation, the 1% concentration proved essentially ineffective, yielding negligible extraction of the polyphenolic fraction, whereas the 5% concentration enabled efficient removal, which became significantly more pronounced at 50% (Fig. 1a). Therefore, the 50% PEG 4000 condition has been selected for the next steps. The extraction process was followed by washing steps with centrifugation (Fig. 1b). The number of washing cycles was evaluated by monitoring the brown color of the supernatant (Fig. 1b). Simultaneously, absorption spectroscopy of the extract exhibited reproducible UV peaks at 226 nm and 322 nm, with a shoulder at 296 nm when analyzed by absorption spectroscopy (Fig. 1c). The wavelength range and the spectral features of these bands clearly distinguish them from typical photosynthetic spectra. Because the 322 nm band showed a strong correlation with the intensity of the brown supernatant, it was used to quantify the phenolic extract (Fig. 1a and b). Four washing steps were sufficient to achieve complete extraction, leaving only a small residual polyphenolic fraction (Fig. 1b). A schematic summary of the steps described in this paragraph is reported in the first part of Table 1.

### Optimized sequential solubilization yields polyphenol-free thylakoid fractions

**Fig. 2:**
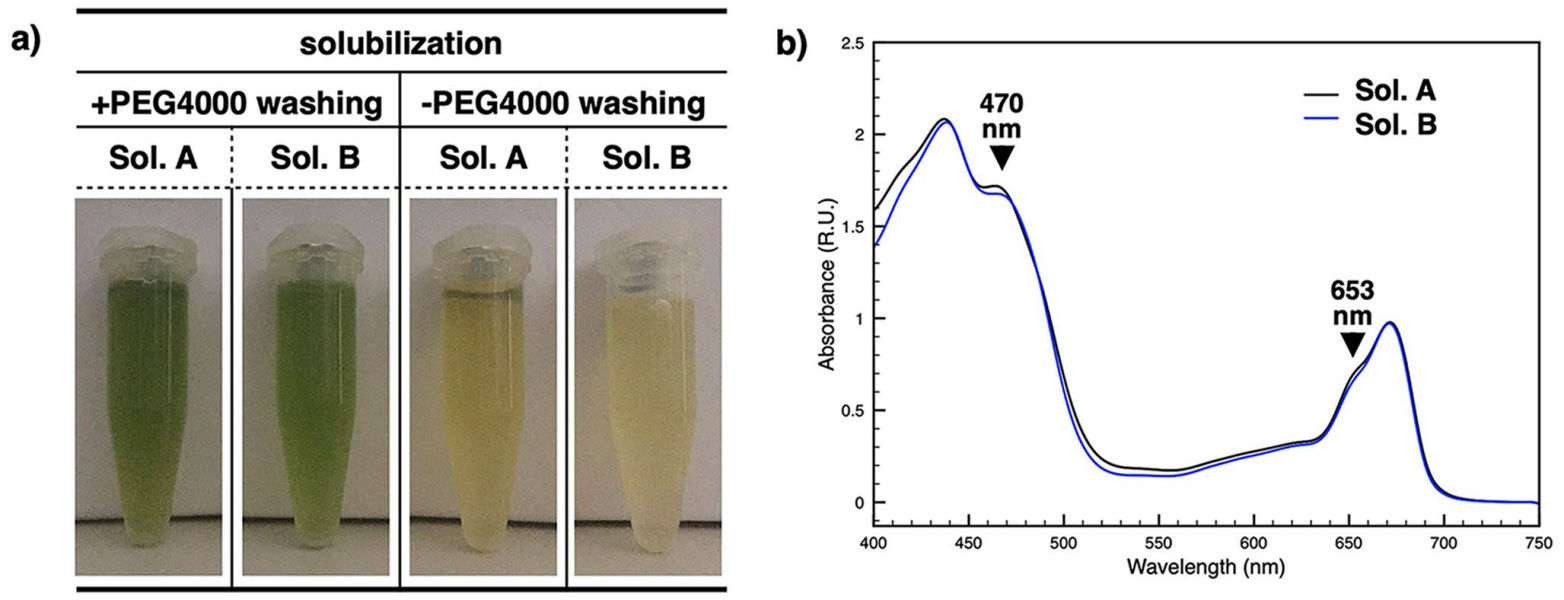
Effect of PEG 4000 pre-treatment on thylakoid solubilization and spectral properties of solubilization pools. Comparison of the two thylakoid solubilization pools A and B (Sol. A and Sol. B) obtained from samples processed with PEG 4000 (top left) or without PEG 4000 pre-treatment (top right). The bottom panel shows the absorption spectra of solubilization pools A and B corresponding to the PEG 4000 treated samples shown in the top-left panel (bottom). The characteristic absorption bands at ~ 470 nm and ~ 653 nm associated with the LHCs are indicated by black arrows

The resulting pellet, effectively depleted of polyphenolic compounds but still containing debris and unbroken fragments, was subjected to direct detergent solubilization instead of passing through intermediated steps of filtration and centrifugation at slow speed, fastening the overall procedure. In a first, direct solubilization step, only freely exposed thylakoid membranes are expected to be accessible to detergent extraction, whereas larger debris and unbroken fragments remain mostly unaffected. The pool resulting from the first solubilization (Solubilization A) was recovered and subsequently characterized. Visual inspection revealed no residual brown–yellow coloration showing an intense green color (Fig. 2a, Sol. A, + PEG4000 washing), and the absorption spectra showed the expected features for photosynthetic complexes while lacking the spectral signature of polyphenolic contamination (Fig. 2b, Sol. A; see Fig. 1c as reference for polyphenolic signature). A parallel solubilization performed on samples that were not pretreated to remove polyphenols yielded negligible material, resulting in an unsuccessful solubilization (Fig. 2a, Sol. A, - PEG4000 washing). This highlights the positive impact of polyphenols on cohesion and structural integrity of the plant tissues, influencing both their stability when subjected to mechanical stress and the recalcitrance of thylakoid membranes to detergents solubilization. Next, we assessed whether additional photosynthetic complexes could be recovered from the remaining pellet through a second solubilization step. This second solubilization produced an additional membrane fraction (Solubilization B) that showed similar characteristics to the ones observed for the first extraction (Fig. 2a, Sol. B, + PEG4000 washing and Fig. 2b, Sol. B). For thylakoids obtained from samples that had not undergone preliminary polyphenol depletion, the second solubilization was also unsuccessful, as already observed for the first solubilization (Fig. 2a, Sol. B, - PEG4000 washing). A schematic summary of the steps described in this paragraph is reported in the second part of Table 1.

### PAGE of solubilized *P. oceanica* thylakoids requires optimized, harsher denaturing conditions

**Fig. 3:**
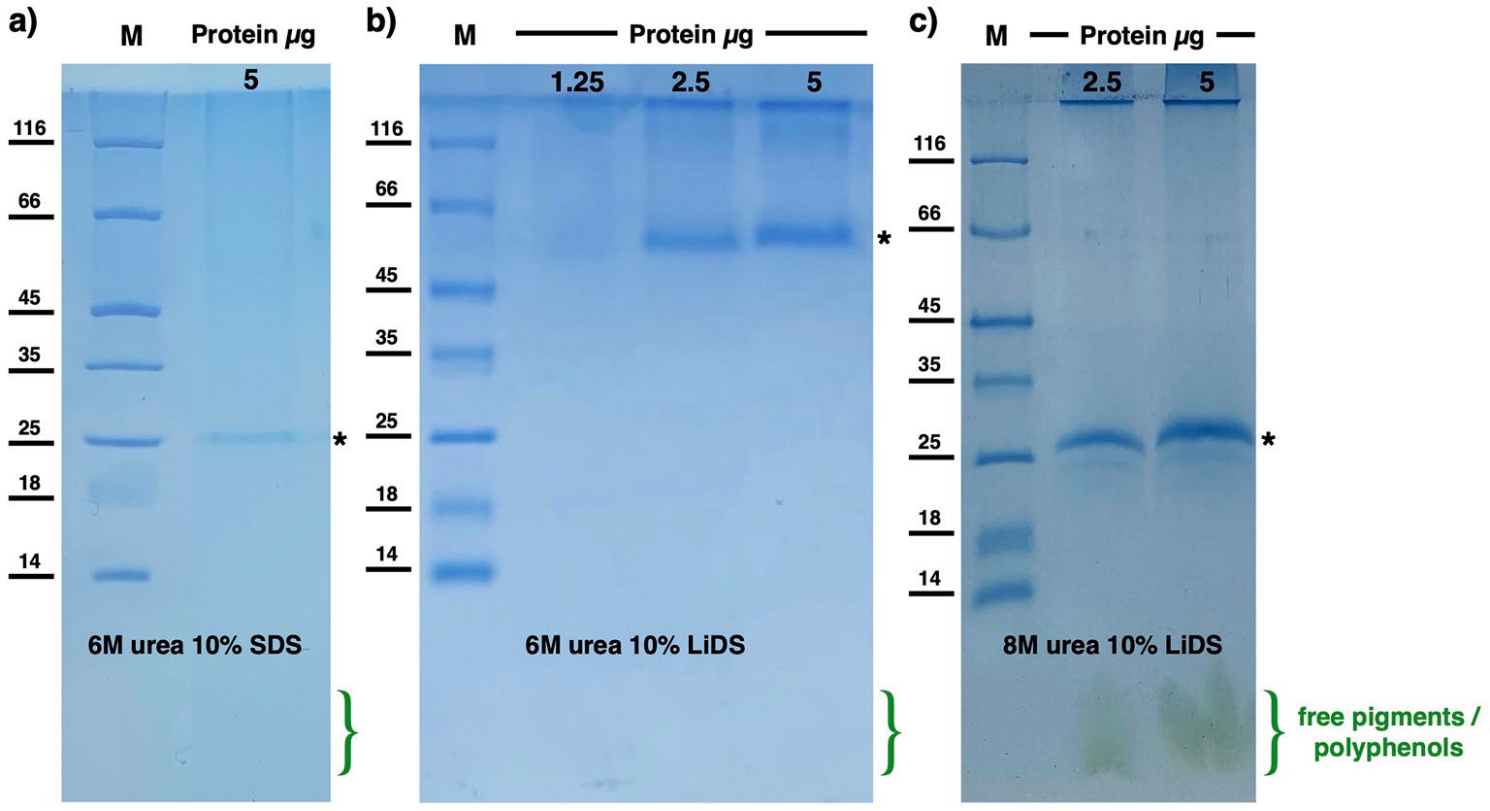
Denaturing electrophoresis of solubilization A of *P. oceanica* thylakoids. The figure shows the behavior of the sample under different PAGE conditions. **a**) Standard gel with 6 M urea and SDS, loaded with ~ 5 µg of protein, showing markedly lower band intensity than expected. The sample exhibits impaired entry into the gel and loss during migration, resulting in reduced signal intensity. The absence of released pigments at the migration front is also consistent with incomplete denaturation; **b**) modified gel with 6 M urea and LiDS, showing improved bands intensity but altered migration and partially diffused bands. The combination of these features, together with the absence of released pigments at the migration front, is consistent with incomplete denaturation; **c**) improved modified gel with 8 M urea and LiDS, showing complete denaturation, as evidenced by a sharp band at the expected apparent mass of ~ 25 kDa, corresponding to LHCs proteins, one of the main components of thylakoid extracts, and the presence of a migration front containing pigments. The amount of loaded sample is expressed in micrograms. M indicates the molecular marker. The asterisk indicates the main band (LHCs) used for monitoring migration/denaturation. Free pigments and residual polyphenols in the migration front are indicated

As an initial biochemical assessment, the solubilized fraction was analyzed by PAGE under denaturing conditions (Fig. 3 and Fig. S1) while native electrophoresis resulted in unsuccessful separation (data not shown). In denaturing electrophoresis, the material did not show canonical or reproducible migration using the standard SDS–PAGE protocol (6 M urea, SDS; Fig. 3a). Instead, samples showed non-reproducible band patterns, variable stain intensities, and in some cases did not enter the gel smoothly, with material partially retained at the bottom of the loading wells. In addition, no released pigments were detected at the migration front. Together, these features indicate incomplete denaturation under standard denaturing conditions. As an example, Fig. 3a shows the separation of 5 µg of total protein from the solubilization A pool. The intensity of the light-harvesting complexes (LHCs), which are signature components of thylakoids and normally migrate at ~ 25 kDa, is noticeably lower than expected. Therefore, an optimized denaturing protocol first substituting SDS with lithium dodecyl sulfate (LiDS; Fig. 3b), then further implemented by employing 8 M urea with LiDS was adopted. These two modifications resulted in efficient migration with respect to the canonical denaturing electrophoresis protocols, as highlighted by the presence of sharp protein bands around 20–30 kDa (LHCs) and released pigments on the migration front (Fig. 3c and Fig. S1). The exclusive visualization of these bands at this stage should not be interpreted as selective enrichment of LHCs but rather reflects the intrinsic dilution of the extract obtained after the polyphenol-removal and solubilization steps. Accordingly, only the most abundant complexes (LHCs) reach clearly detectable levels on denaturing PAGE and, for the same reason, dominate with their spectral signatures at 470 nm and 653 nm in the spectra shown in Fig. 2. Other photosynthetic complexes are present but fall below the detection threshold as also evidenced when loading higher amount of protein in denaturing gels (Fig. S1).

### Dominant components are enriched by preparative chromatography

**Fig. 4:**
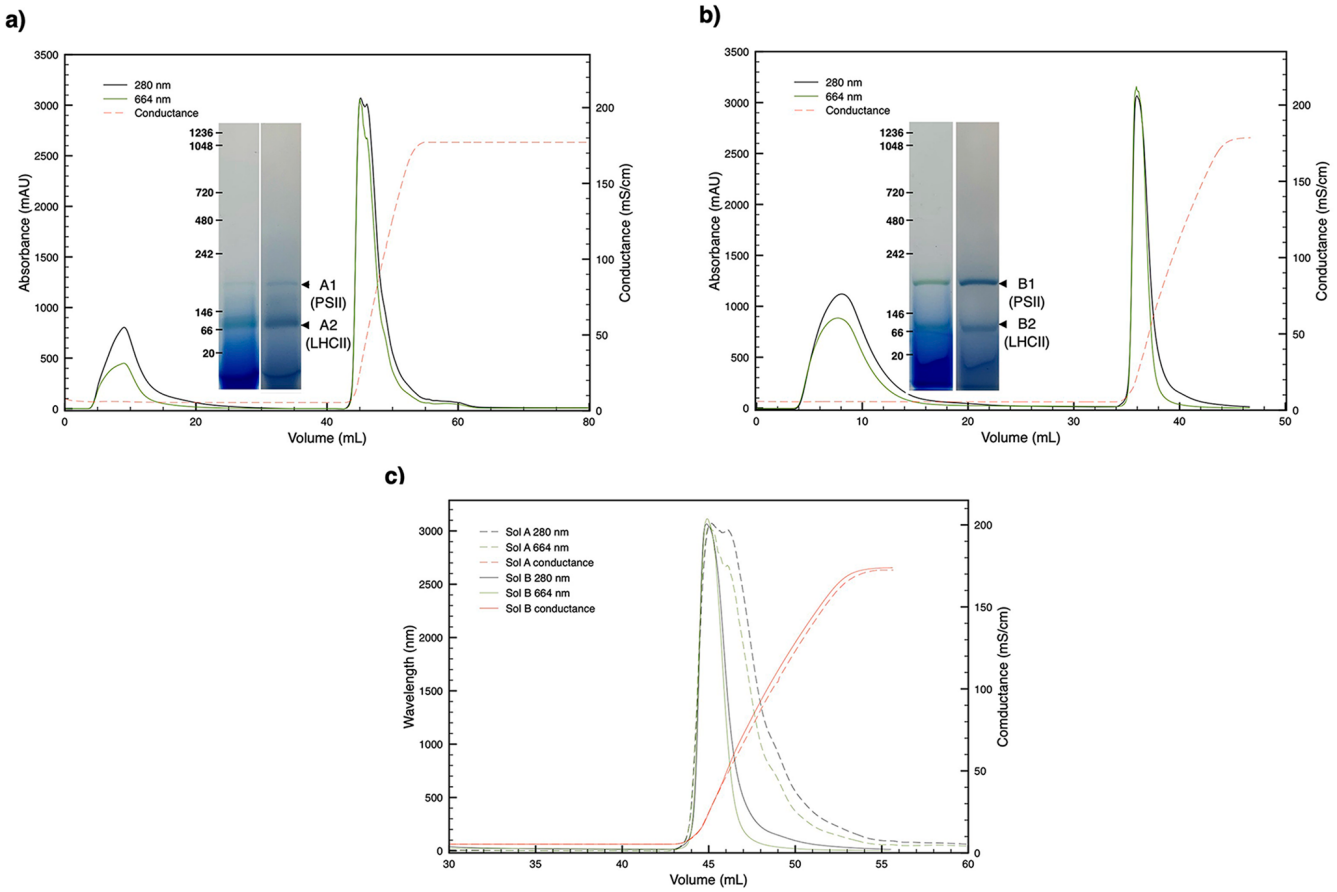
Anion-exchange chromatography of solubilization pools A and B. **a**) Anion-exchange chromatography of the Solubilization A pool resulted in two elution peaks; native PAGE analysis of the collected pool of fractions unstained and stained (inset) shows a dominant band at ~ 70 kDa. **b**) Chromatography of the Solubilization B pool yielded a single, well-defined peak; native PAGE analysis of the collected pool of fractions (inset), here shown both unstained and stained, shows a major band at ~ 200 kDa. **c**) When the chromatographic profiles are aligned, the first peak from Sol. A and the single peak from Sol. B overlap, indicating that both solubilization steps recover complexes with partially shared charge properties

After preliminary characterization, both solubilization pools were subjected to preparative anion-exchange chromatography (Fig. 4 and Fig. S2). Each showed a distinct separation profile. The solubilization A resolved into two partially overlapping peaks (Fig. 4a), whereas the solubilization B produced a single peak (Fig. 4b). Elution profiles were compared by aligning them to the conductance variation profile (Fig. 4c), since both procedures followed the same geometry and gradient parameters. This approach revealed that the solubilization B peak aligns with the first peak of the solubilization A, so that the pool of the solubilization B appears to be enriched in one of the A components (Fig. 4c). Native electrophoresis further confirmed differences between the pools, yet in both cases two bands were detected, one of which was predominant (insets Fig. 4a and b). Specifically, a single pool including both overlapping peaks from solubilization A exhibited a dominant band at approximately ~ 70 kDa, consistent with putative LHCII trimers, and a minor band at ~ 200 kDa, compatible with PSII monomers and/or partially assembled PSII forms. The solubilization B, instead, showed a similar pattern but with inverted relative intensities (insets Fig. 4a and b), suggesting that the first peak of A and the peak of B are PSII-related.

### The pools’ components are identified and annotated as subunits of PSII and LHCII complexes

Next, the native bands obtained by BN-PAGE from the chromatographic pools of solubilization A (bands A1 and A2) and solubilization B (bands B1 and B2) were excised from the gel and subjected to in-gel digestion (insets Fig. 4a and b). The resulting peptides were analyzed by LC–MS/MS to determine the protein composition of each band. This analysis enabled the assignment of the dominant complexes in each band. Although all bands showed heterogeneous profiles, key characteristics, such as their apparent molecular weights on BN-PAGE and the representativity of the identified entries in MS, allowed a confident assignment for each band. As expected, based on the apparent band mass of ~ 200 kDa, typical for PSII monomers, bands A1 and B1 exhibited highly similar peptide profiles. Together, they are composed of a full set of major PSII core subunits, including PsbA, PsbB, PsbC, PsbD, PsbE, PsbH, PsbL, and PsbO. While these seven subunits are among the top eight MS hits, the highest scoring hit corresponded to LHCII (identified through the homologs CB27_TOBAC and CB22_MAIZE, equivalent to Lhcb1.3 and Lhcb1.5, on the A1 and B1 bands, respectively; Table S1). This likely reflects their partial co-migration, nonspecific interactions, and, based on the behavior of these samples during electrophoretic separation, aggregation or precipitation, consistent with the high abundance of LHCII in thylakoids. This interpretation is further supported by the absence of other LHCII isoforms with high scores, which are normally required to form stable LHCII trimers. Conversely, bands A2 and B2, with an apparent mass of ~ 70 kDa consistent with LHCII trimers, were enriched in chlorophyll-binding proteins.

The proteomic results are summarized in Table 2. Based on the identified subunit composition together with the apparent molecular mass inferred from the BN-PAGE, bands A1/B1 were enriched in monomeric PSII core complex, while bands A2/B2 represent trimeric LHCII.

**Table 2:** Mass spectrometry results for each BN-PAGE band shown in the insets of Fig. 4. For each band (A1, B1, A2, B2), the table reports the closest homologous protein identified in the MS search, including its gene name, protein name, associated photosynthetic complex, and source organism. For each entry, the nearest *Arabidopsis Thaliana* homolog and its molecular mass are indicated and used to assign names to the proteins identified in *P. oceanica* (see Table 3 for details). The first entry in A1 and B1, the LHCII peptide (shown in red), was considered a contaminant as it is likely the result of partial co-migration or nonspecific interactions. This interpretation is consistent with the high abundance of LHCII proteins in thylakoid membranes and their known tendency to aggregate or precipitate during electrophoresis. The MASCOT score and the score normalized per mass unit are provided; the most abundant entry (as part of PSII for bands A1 and B1, and LHCII for band A2 and B2) was set to 1, and all others are expressed as indices relative to it. A cutoff index of 0.4 was applied for the lower-ranking entries, yielding PSII subunits as the main components of A1 and B1, and LHCII proteins as the dominant components of A2 and B2. Additional details, including the complete list of identified proteins, are provided in Table S1

BN-PAGE band	LC-MS/MS entry	Gene	Protein	Complex	Organism	Homologous entry in A. thaliana	Mass (kDa)	*P. ocenica* entry name	MASCOT score	Quantitative presence (weighted)
A1	P27491	lhcb1.3	LHCB1.3	LHCII	*Nicotiana tabacum*	P04778	28.33	CB1C_POSOC	1942	2.273
	Q70XY9	psbE	PsbE	PSIIcc	*Amborella trichopoda*	P56779	9.39	PSBE_POSOC	283	1.000
	Q3V554	psbA	PsbA (D1)	PSIIcc	*Acorus calamus*	P83755	38.94	PSBA_POSOC	928	0.790
	Q7J1C4	psbL	PsbL	PSIIcc	*Acorus calamus*	P60129	4.47	PSBL_POSOC	105	0.780
	Q4FFP4	psbD	PsbD (D2)	PSIIcc	*Acorus calamus*	P56761	39.55	PSBD_POSOC	866	0.730
	P19819	psbB	PsbB (CP47)	PSIIcc	*Oenothera argillicola*	P56777	56.04	PSBB_POSOC	1136	0.670
	P84718	psbO	PsbO	PSIIcc	*Pinus strobus*	Q9S841	35.02	PSBO2_POSOC	180	0.490
	Q3BAP4	psbC	PsbC (CP43)	PSIIcc	*Phalaenopsis aphrodite*	P56778	51.87	PSBC_POSOC	757	0.490
B1	P06671	lhcb1.5	LHCB1.5	LHCII	*Zea mays*	Q39141	28.05	CB1E_POSOC	4639	5.536
	Q70XY9	psbE	PsbE	PSIIcc	*Amborella trichopoda*	P56779	9.39	PSBE_POSOC	281	1.000
	Q4FFP4	psbD	PsbD (D2)	PSIIcc	*Acorus calamus*	P56761	39.55	PSBD_POSOC	794	0.670
	P19819	psbB	PsbB (CP47)	PSIIcc	*Oenothera argillicola*	P56777	56.04	PSBB_POSOC	944	0.560
	P84718	psbO	PsbO	PSIIcc	*Pinus strobus*	Q9S841	35.02	PSBO2_POSOC	203	0.550
	Q7J1C4	psbL	PsbL	PSIIcc	*Acorus calamus*	P60129	4.47	PSBL_POSOC	68	0.510
	Q3V554	psbA	PsbA (D1)	PSIIcc	*Acorus calamus*	P83755	38.94	PSBA_POSOC	548	0.470
	Q9SDMI	lhca1	LHCA1	LHCI	*Hordeum vulgare*	Q01667	26	CAB6_POSOC	348	0.440
A2	P06671	lhcb1.5	LHCB1.5	LHCII	*Zea mays*	Q39141	28.05	CB1E_POSOC	6465	1.000
B2	P06671	lhcb1.5	LHCB1.5	LHCII	*Zea mays*	Q39141	28.05	CB1E_POSOC	5105	1.000
	P27524	lhcb6	CP24	PSIIsc	*Solanum lycopersicum*	Q9LMQ2	27.52	CP24_POSOC	2022	0.410

As shown in Table 2, peptide identification by LC–MS/MS was challenged by the fact that *P. oceanica* does not yet have a curated proteome, and only a very limited number of protein entries for this species are available in UniProt. Therefore, direct species-specific annotation was not possible. To address this, each MS/MS peptide spectrum was first searched by homology against well-annotated photosynthetic organisms, allowing us to identify the most similar protein entries and their associated gene identifiers. These annotated sequences were then used as reference templates to query the existing *P. oceanica* genomic and transcriptomic resources. In this way, we could recover the most plausible native coding sequence for each detected PSII and LHCII subunit. Thus, MS data not only confirmed the identity of the isolated complexes but also enabled inference and refinement of *P. oceanica* protein annotations, effectively extending the current proteomic information available for this species (Table 3).

**Table 3:** Annotation of *P. oceanica* sequences based on LC-MS/MS–identified homologous proteins. Using the MS-identified entries corresponding to homologs of protein components in the *P. oceanica* samples, the respective *P. oceanica* gene locations and inferred protein sequences were retrieved. In the annotation, each gene name was assigned according to the naming code of the homolog identified in the model organism *Arabidopsis Thaliana* (see Table 2 for details)

LC-MS/MS entry name	*P. oceanica* entry name	Gene location	Protein sequence
PSBA_ACOCL	PSBA_POSOC	> JAZBRT010000221.1:12140848–12,141,146 Posidonia oceanica cultivar Po_2 chromosome 2 Chr02_contig_88 PARTIAL SEQUENCE	LYGGGPCDLNVLHFLLGVACCLGHVGELGFPIVVATVVFLIYPISQRSFYDIMHLRIFDTFNFMIVFQTAHNILMHPFLMLGIVDVFSSSLVAPMQGSFVPSTLISGATENKSANEGYRFDQEEEACNIAVEATHGYFGRLIFQYASFSNSHFLQFSLSAWPMVGTFTMLVLATLIFYNFPYPLGLWVLSLLLQPVIEIIYHANLGMKVMHECNAHNFPLDLTVVEAPSING
PSBB_OENAR	PSBB_POSOC	> JAZBRS010000014.1:76157605–76,158,076 Posidonia oceanica cultivar Po_2 chromosome 1 Chr01_contig_14 PARTIAL SEQUENCE	FDPFVPGGIASHHIAVGMLGILAGLFHLSVRLPQRLYKGLRMGNIETVLSSSITAVFFAAFVVVGTMWYGSATTPIKLFGPTHYWDQGYFQQEIYRRVVTRLAENLSLLEAWSKIPEKLAFYDYIDNNLAKGGLFRAGSMDIGDGIAVGWLGHPIF
PSBC_PHAAO	PSBC_POSOC	> JAZBRS010000047.1:33672568–33,675,041 Posidonia oceanica cultivar Po_2 chromosome 5 Chr05_contig_2 MISSING N-TERM	IDNQTVVCLGNSMWCSEMVEVVQEDNYDYNPWIYQRGKFIYYGLVTKGSFCFCGLVRSIALSLCLFRSRGFGSRVQLLLHGYTHGLASSYLEGCNFLTAAVSTLCCYYGAPEAQGDFTHWCQLGGLWTFVALHGAFGPMGFMLRQFELVRSMLRPSNAIAFSDPIAIFLFRYSFIHWVSLVGFLRLVLVRRYFDASFSSKDFIIGHIHFIWELPENVLLCCALFMVLCCRKYFIRRWRRKYIPRFPNSSRRDLFNGHCPLLVTNLRGCFFQTLVTFLYVICTSNRFMDERSWSNRSGSEPTCLLRFAGNPCSGGSIRDFLHQKNILLNEGIRAWMAAQDQPHENLIFPEEVLPHGNALWNFSFSWSPRNHWFRLVGRECQTYQFVTTWGPRSPCRINCILGKSNEPISGPFRTREAHVRIRIDFTSPSNYSRLGYRSKWGSYRHVSLNFLRSLRLWRYLSASWVDSGIFSILWLSMERKKDHNFRYSLNFVSYRCFSSSISPFILVAYMIPGLLGVEMEKLPTPLAQVLYLVIYNLPLGGEDGLLVWTIKILEAMYGVPFVYLVEFGISPSPLHGLAVHLSGLERLTCLIVLLYLFFDFIACCFVWFNNTAYPSEFYGPTGPEAFEAQAFIFLVRDQRLGANIGSAQGPIGLGKYLMRSPTGEIIFGGETMRFWDLCVPWLEPLRGTNGLDLSRLKKDIQPWQERRSTEYMTHAPLGSLNFMGGVATEINAVNYVSPSWLATSHFVLGFFLFVGHLWHAGRARAVAAAFEKGIDRDLEPVLSMTPL
PSBD_ACOCI	PSBD_POSOC	> JAZBRT010000523.1:1075315 to 1,077,407 Posidonia oceanica cultivar Po_2 chromosome 5 Chr05_contig_6 Range 1: 1,075,315 to 1,077,407 MISSING N-TERM	RGFGSRVQLLLHGYTHGLASSYLEGCNFLTAAVSTLCCYYGAPEAQGDFTHWCQLGGLWTFVALHGAFGLMGFMLRQFELVRSMLRPSNAIAFSDPIAIFLFRYSFIHWVSLVGFLRLVLVRRYFDASFSSKDFIIGHIHFIWELPENVLLCCALFMVLCCRKYFIRRWRRKYIPRFPNSSRRDLFNGHCPLLVTNLRGCFFQTLVTFLYVICTSNRFMDERSWSNRSGSEPTCLLRFAGNPCSGGSIRDFLHQKNILLNEGIRAWMAAQDQPHENLIFPEEVLPHGNALWNFSFSWSPRNHWFRLVGRECQTYQFVTTWGPRSPCRINCILGKSNEPISGPFRTREAHVRIRIDFTSPSNYSRLGYRSKWGSYRHVSLNFLRSLRLWRYLSASWVDSGIFSILWLSMERKKDHNFRYSLNFVSYRCFSSSISPFILVAYMIPGLLGVEMEKLPTPLAQVLYLVIYNLPLGGEDGLLVWTIKILEAMYGVPFVYLVEFGISPSPLHGLAVHLSGLERLTCLIVLLYLFFDFIACCFVWFNNTAYPSEFYGPTGPEAFQAQAFIFLVRDQRLGANIGSAQGPIGLGKYLMRSPTGEIIFGGETMRFWDLCVPWLEPLRGTNGLDLSRLKKDIQPWQERRSTEYMTHAPLGSLNFMGGVATEINAVN
PSBE_AMBTC	PSBE_POSOC	> JAZBRS010000020.1:78831118–78,831,366 Posidonia oceanica cultivar Po_2 chromosome 2 Chr02_contig_3	MSGSTGERSFADIITSIRYWVIHSITIPSLFIAGWLFVSTGLAYDVFGSPRPNEYFTESRQGIPLITGRFDPLEQLDEFSRSF
PSBH_PIPCE	PSBH_POSOC	> JAZBRS010000055.1:48176223–48,176,384 Posidonia oceanica cultivar Po_2 chromosome 6 Chr06_contig_1	MATQTVEGSSRSGPRRTVIGDLLKPLNSEYGKVAPGWGTTPFMGVAMALFAIFL
PSBL_ACOCL	PSBL_POSOC	> JAZBRS010000020.1:78831521–78,831,661 Posidonia oceanica cultivar Po_2 chromosome 2 Chr02_contig_3	MTQSNPNEQNVELNRTSLYWGLLLIFVLAVLFSNYFFNEIERKRI
PSBO_PINST	PSBO2_POSOC	> JAZBRT010000235.1:6662594–6,663,352 Posidonia oceanica cultivar Po_2 chromosome 3 Chr03_contig_1	MEVKGTGTANQCPTIDGGSESFPFKAGKYNMKNLCLEPTSFTVKAEGVAKNSPLEFQKTKLMTRLTYTLSDIEGPFEISSNGKVKFEEKDGIDYAAVTVQLPGGERVPFLFTIKQLVASGTPDKIEGSFLVPSYRGSSFLDPKGRGGSTGYDNAVALPAGGRGDEEVLAKENIKDNTSSTGKISFTVTKSKPQTGEVIGVFESIQPSDTDLGAKVPKDVKIQGIWYAQLD
CA4_ARATH	CA4_POSOC	> JAZBRS010000013.1:c29645498-29644677 Posidonia oceanica cultivar Po_2 chromosome 1 Chr01_contig_13	MATFTAQASSTAFQSCRSKSRFLSGTSTKLPRHFSVTAAPTTTTTSTSLKVEAKKGEWLPGLASPNYLNGRFVLESFFTNSFQCNVCHQSNQLMTYNGFDVSMQCSSWNPSLSINCELDVRYSLPGDNGFDPLGLGEDPESLKWFVQAELQNGRWAMLAVAGVLLPEVLTKIGIINVPEWYDAGKSEYFASSSTLFVIEFILFHYVEIRRWQDIKNPGCVNQDPIFKNYSLPPGEVGYPGGIFNPLNFAPSLEAKEKEIANGRCNSCSYC
CB2_MALDO	CB1C_POSOC	> JAZBRS010000031.1:2890946–2,891,585 Posidonia oceanica cultivar Po_2 chromosome 4 Chr04_contig_1	MLGTLGCVFPELLSRNGVKFGEAVWFKAGSQIFSDGGLDYLGNPSLVHAQSILAIWACQVILMGAVEGYRIAGGPLGEVVDPLYPGGSFDPLGLAEDPEAFAELKVKEIKNGRLAMFSMFGFYVQAIVTGKGPLENLADHLADPVNNNAWAYATNFVPGK
CB3_ARATH	CB3_POSOC	> JAZBRS010000076.1:3492620–3,493,135 Posidonia oceanica cultivar Po_2 chromosome 10 Chr10_contig_1	MLGTFGCITPEVLAKWLKVQFKEPVWFKAGSQIFSAGGLDYLGNPNLVHAQSILAVLGIQVILMGLVESYRINGLPGVGEGNDLYPGGQYFDPLGLADDPVTFAELQVKEIKNGRLAMFSMFGFYVQAIVTGKGPLENLLDHLDNPVANNAWAYATKFVPGA
CB4A_SOLLC	CP24_POSOC	> JAZBRS010000023.1:18516859–18,517,410 Posidonia oceanica cultivar Po_2 chromosome 2 Chr02_contig_6 MISSING N-TERM	RLPGDYGFDPLGLGKDPAFLKWYREAELIHGRWAMAAVLGIFVGQAYSGIPWFEAGADPGAVAPFSFGTLLGTQLLLMGWVEGKRWVDFFNPESQSVEWATPWSRTAENFANATGDQGYPGGKFFDPLSLAGTVKNGVYVPDMKKLDRLKLAEIKHARIAMLAMLTFYFEAGQGKTPLGA LGL
CB5_ARATH	CB5_POSOC	> JAZBRT010000397.1:1540298–1,540,918 Posidonia oceanica cultivar Po_2 chromosome 4 Chr04_contig_2 MISSING N-TERM	SPWYGPDRVKYLGPLSGEAPSYLTGEFPGDYGWDTAGLSADPETFAKNRELEVIHCRWAMLGTLGCVFPELLSRNGVKFGEAVWFKAGSQIFSDGGLDYLGNPSLVHAQSILAIWACQVILMGAVEGYRIAGGPLGEVVDPLYPGGSFDPLGLAEDPEAFAELKVKEIKNGRLAMFSMFGFYVQAIVTGKGPLENLADHLADPVNNN
CB121_HORVU	CAB6_POSOC	> JAZBRS010000057.1:12152671–12,153,226 Posidonia oceanica cultivar Po_2 chromosome 6 Chr06_contig_3 PARTIAL SEQUENCE	AARDLGAGGVGTRELGEGPGVGSGARWPSHLLGQPSPVGHSPHHPRRRVHHHRLRRAAAHGEGPREEEVPRWRLPLGLLQGPRQVTGVQGEGDQERVEYYYYLRQHHQYSLRMYICVCFIKVHGFALAGRLALLAFVGFCVQQSAYPGTGPLENLATHLADPWHNNIGDVVIPRSIFP
CB22_MAIZE	CB1E_POSOC	> JAZBRS010000031.1:3412776–3,413,577 Posidonia oceanica cultivar Po_2 chromosome 4 Chr04_contig_1	MAATMALSSPSLAGKAVKLAPSASEILGAGRVTVRSTIKSKAAPGSPWYGPDRVKYLGPLSGEAPSYLTGEFPGDYGWDTAGLSADPETFAKNRELEVIHCRWAMLGTLGCVFPELLSRNGVKFGEAVWFKAGSQIFSDGGLDYLGNPSLVHAQSILAIWACQVILMGAVEGYRIAGGPLGEVVDPLYPGGSFDPLGLAEDPEAFAELKVKEIKNGRLAMFSMFGFYVQAIVTGKGPLENLADHLADPVNNNAWAYATNFVPGK
CB23_SOLLC	CB3_POSOC	> JAZBRS010000076.1:3492621–3,493,134 Posidonia oceanica cultivar Po_2 chromosome 10 Chr10_contig_1	MRRGTVKPPISGSPWYGRDRVKYLGPFSGEAPSYLTGQFPGDYGWDTAGLSADPETFAKNRELEVIHARWAMLGTLGCVFPELLARNGVKFGEAVWFKAGSQIFSEGGLDYLGNPSLVHAQSILAIWATQVILMGAVEGYRVAGGPLGDVDDPLYPGGSFDPLGLADDPEAFAELKVKEIKNGRLAMFSMFGFYVQAIVTGKGPLENLADHLADPVNNNAWAYATNFVPG
CB27_TOBAC	CB1C_POSOC	> JAZBRS010000031.1:2865243–2,866,037 Posidonia oceanica cultivar Po_2 chromosome 4 Chr04_contig_1	MAATMALSSPSLAGKAVKLAPSASETLGAGRVTMRSTIKSKAVPGSPWYGPDRVKYLGPLSGEAPSYLTGEFPGDYGWDTAGLSADPETFAKNRELEVIHCRWAMLGTLGCVFPELLSRNGVKFGEAVWFKAGSQIFSDGGLDYLGNPSLVHAQSILAIWACQVILMGAVEGYRIAGGPLGEVVDPLYPGGSFDPLGLAEDPEAFAELKVKEIKNGRLAMFSMFGFYVQAIVTGKGPLENLADHLADPVNNNAWAYATNFVPGK

### Distinctive absorption signatures point to LHCII- and PSII-enriched samples

**Fig. 5:**
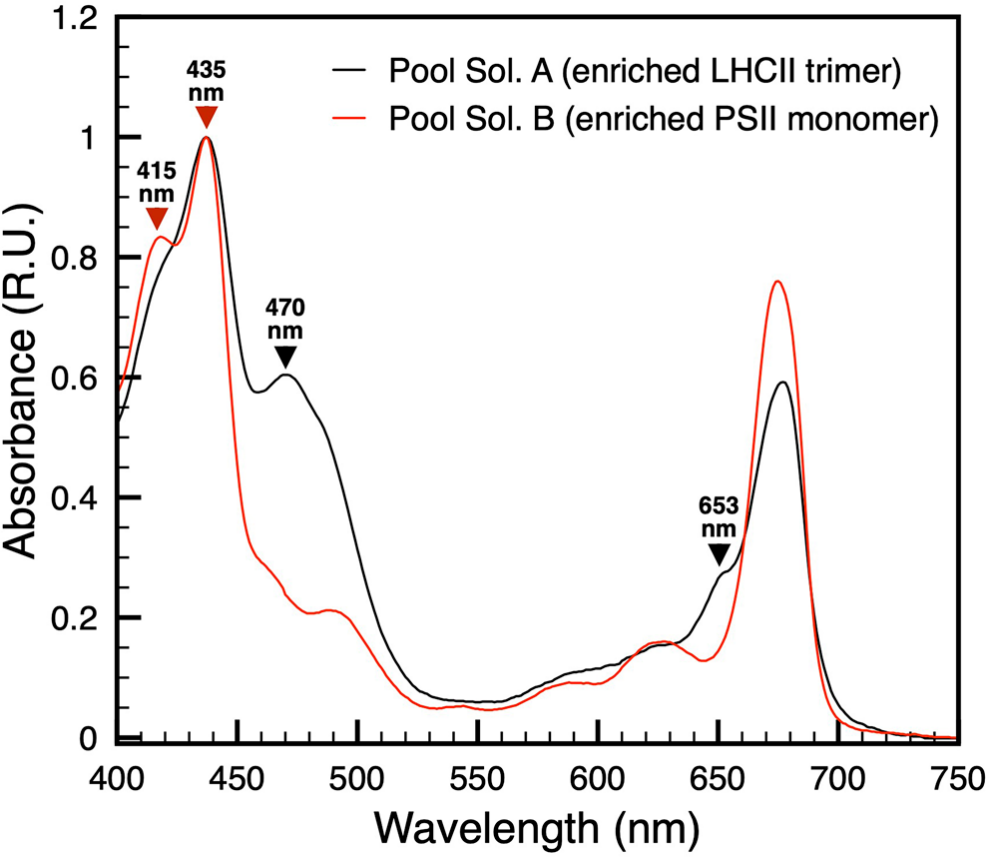
Absorption spectra of chromatography-resolved thylakoid pools The absorption spectra of the two chromatographic pools show distinct features. Solubilization A (black) shows a peak–shoulder at ~ 653 nm in the Q_y_ region, indicative of chlorophyll b (black arrow). In the Soret region, a peak at ~ 470 nm reflects carotenoid contributions consistent with LHCII enrichment (black arrow). Solubilization B (red) in the Soret region exhibits peaks at ~ 415 nm (red arrow - pheophytin and/or cytochrome b559 heme) and ~ 435 nm (red arrow -chlorophyll a), consistent with enrichment in PSII complexes

The two chromatographic pools were eventually characterized by absorption spectroscopy (Fig. 5). The samples showed distinct spectral profiles, confirming enrichment in different photosynthetic components. The absorption spectra obtained from the chromatographic pool A exhibits a peak–shoulder at ~ 653 nm in the Q_y_-region, indicative of chlorophyll *b* and characteristic of LHCII, along with a main peak at ~ 677 nm corresponding to chlorophyll *a*. In the Soret region, besides the chlorophyll peak at ~ 435 nm, a prominent peak at ~ 470 nm was observed, reflecting the contribution of carotenoids and further supporting the predominance of LHCII. In contrast, the chromatographic pool B exhibits features typical of PSII complexes: the Q_y_-region lacked the chlorophyll *b* shoulder at 653 nm, and in the Soret region a distinct peak at ~ 415 nm, attributable to pheophytin and/or cytochrome b559 heme, appeared alongside the chlorophyll *a* Soret maximum at ~ 435 nm, with no evident carotenoid-associated band at 470 nm. Taken together, these spectral characteristics confirmed that pool A is enriched in LHCII trimers, whereas pool B is enriched in PSII complexes.

## Discussion

It is well established that, in higher plants, the architecture of the photosynthetic apparatus is widely conserved, whereas its functional properties are remarkably plastic. Evolution has shaped this system so that different species, while retaining the same core structural framework, have adapted their photosynthetic machinery to operate optimally within the specific environmental “light niches” they occupy (Nelson and Yocum 2006; Ruban 2016; Blankenship, 2002). Thus, across plant lineages, the photosynthetic apparatus can be viewed as a conserved platform whose functional tuning, in terms of light quality, light intensity, and regulatory responses, diverges according to ecological pressures.

In this context, *Posidonia oceanica* represents an extreme case. As a marine angiosperm living entirely submerged, it is exposed to a light environment profoundly different from terrestrial habitats: light quantity is strongly attenuated with depth, and light quality is progressively shifted toward the blue region of the spectrum (Kirk 2011). Despite these constraints, *P. oceanica* exhibits remarkable photophysiological plasticity, being able to colonize habitats ranging from shallow waters near the surface to depths of 40–50 m, depending on water transparency (Dattolo et al. 2013; Procaccini et al. 2017; Koopmans et al. 2020; Madonia et al. 2021). This implies that its photosynthetic apparatus must operate efficiently across a wide and variable range of irradiance and spectral composition.

Accordingly, in *P. oceanica* the plasticity of the photosynthetic apparatus likely co-evolved with the ability to colonize depth-variable environments. Increasing depth imposes strong selective pressures; in fact, light intensity is progressively reduced, its spectral composition shifts toward blue wavelengths, and hydrodynamic forces increase. Successful colonization, therefore, requires not only photophysiological flexibility, but also extensive structural adaptations of the entire plant, particularly the leaf. In *P. oceanica*, leaves are thin, elongated, coriaceous and exceptionally rich in polyphenolic compounds (Benito-González et al. 2019; Astudillo-Pascual et al. 2021). These features, while advantageous in the marine environment, are significantly challenging for a complete biochemical characterization. The mechanical toughness of the tissue complicates fragmentation and homogenization, and the high polyphenol content interferes with downstream isolation of stable and pure thylakoid membranes, due to their high reactivity with both proteins and membrane surfaces (Sęczyk et al. 2019), especially for spectroscopic applications, where these secondary metabolites can strongly absorb light or interact with pigments (Sęczyk et al. 2019).

In fact, in phenolic-rich plants such as *Posidonia oceanica* and many gymnosperms, polyphenols interact with proteins through both non-covalent and covalent chemistry. Hydroxyl-rich polyphenols, including tannins, form reversible hydrogen-bonded and π–π networks with protein backbones and Pro-rich regions (Adamczyk et al. 2017; Ozdal et al. 2013). Under oxidative conditions, polyphenols can be converted physiologically to quinones that covalently cross-link nucleophilic residues (Lys, Cys, His), generating protein–polyphenol networks (Boeckx et al. 2015). In membrane proteins, such reactions are expected to increase membranes rigidity and reduce protein solubility by limiting detergent accessibility, making membranes largely resistant to detergent-mediated solubilization (Bag et al. 2021; Charras et al. 2024). PEG can disrupt such phenolic networks by forming hydrogen bonds with polyphenols, increasing their solubility and competing with proteins for polyphenol binding sites (Seker et al. 2019). In extraction protocols for *P. oceanica* and other phenolic-rich species, PEG in buffers neutralizes polyphenol reactivity and improves protein and thylakoid recovery (Bag et al. 2021; Charras et al. 2024). To overcome the above-mentioned limitation, in recent years, several studies have focused on optimizing the isolation and characterization of thylakoids and photosynthetic complexes from plants adapted to extreme or atypical environments. The first recent work in this area was carried out by Bag and coworkers (2021) on gymnosperms, particularly on spruce and pine (*Picea abies* and *Pinus sylvestris*, respectively). In these species, adaptation to cold and dry environments is associated with anatomical modifications of the leaves and a marked increase in tannins and other polyphenols, features that are also shared in the adaptation strategies of *P. oceanica*. Bag et al. (2021) described a detailed procedure that includes sorbitol, PEG 6000, ascorbic acid, sodium fluoride, and bovine serum albumin to counteract polyphenol interference during thylakoid isolation. More recently, a similar procedure was applied on *P. oceanica*, employing essentially the same key components (Charras et al. 2024) also allowing for a preliminary characterization of PSI samples from this species (Charras-Ferroussier et al. 2025).

In the present work, we essentially applied the procedure previously developed on conifers (Bag et al. 2021), while incorporating subsequent advances reported for *P. oceanica* (Charras et al. 2024). Our goal was to further increase the efficiency through a more direct workflow, a monitored quantification of polyphenol extraction and a subsequent thylakoid differential solubilization, which in this study was performed in two sequential steps (Figs. 1 and 2). In particular, polyphenol extraction was quantified based on the typical absorption properties of the sample, specifically the absorption band at 322 nm, which were consistent with the spectral characteristics of similar samples reported in the literature (Markham 1982; Harborne 1984; Heim et al. 2002; Anouar et al. 2012). These polyphenol extraction steps not only prevent interference from secondary metabolites but also makes the leaf material more accessible, facilitating subsequent and differential membrane extraction. Importantly, thylakoids are solubilized directly from the crude fragmented tissue, without intermediate debris-removal steps (Fig. 2). This strategy minimizes processing time and reduces sample manipulation, both critical factors which can compromise the stability of photosynthetic complexes.

Using this procedure, we obtained solubilized thylakoids in quantities sufficient for the selective isolation of individual photosynthetic complexes by preparative chromatography (Fig. 4). As expected, preliminary characterization of solubilized thylakoids by denaturing electrophoresis revealed a strong dominance of LHCII, driving our focus on its preparative isolation by chromatographic separation. Notably, even at this stage, electrophoretic analysis required specific optimization (Fig. 3), likely due to residual polyphenolic components that, similarly to what is observed during detergent solubilization, interfere with protein denaturation and sample migration by hindering SDS function. This behavior is consistent with reported phenomena in phenolic-rich tissues, where polyphenols do not necessarily induce protein insolubility but rather shield membrane proteins from detergent action, reducing their extractability and impairing electrophoretic separation (Sęczyk et al. 2019). For this reason, a more stringent denaturing protocol was adopted, employing 8 M urea and replacing sodium dodecyl sulfate with lithium dodecyl sulfate (Delepelaire and Chua 1979; Yada et al. 1979). This strategy allowed complete protein denaturation, as evidenced by the migration of the released chlorophylls (green component) and polyphenols (brown component) at the dye front, an effect not observed under standard denaturing electrophoresis conditions (Fig. 3 and Fig. S1).

In this study, we also optimized the use of solubilized thylakoids for the preparative isolation of LHCII, establishing a workflow that can be readily extended to other photosynthetic complexes. This approach enabled a preliminary characterization of the isolated LHCII, confirming its structural trimer stability by native electrophoresis, absorption spectroscopy, and mass spectrometry which eventually allowed identification of its protein composition. Using the same strategy, but working on the second solubilization pool, we successfully applied the chromatography procedure to the isolation of PSII core complexes as well. PSII was isolated and preliminarily characterized, appearing predominantly in its monomeric form in native electrophoresis, consistent with the subunits identified by mass spectrometry (Tables 2 and 3, Table S1).

**Fig. 6:**
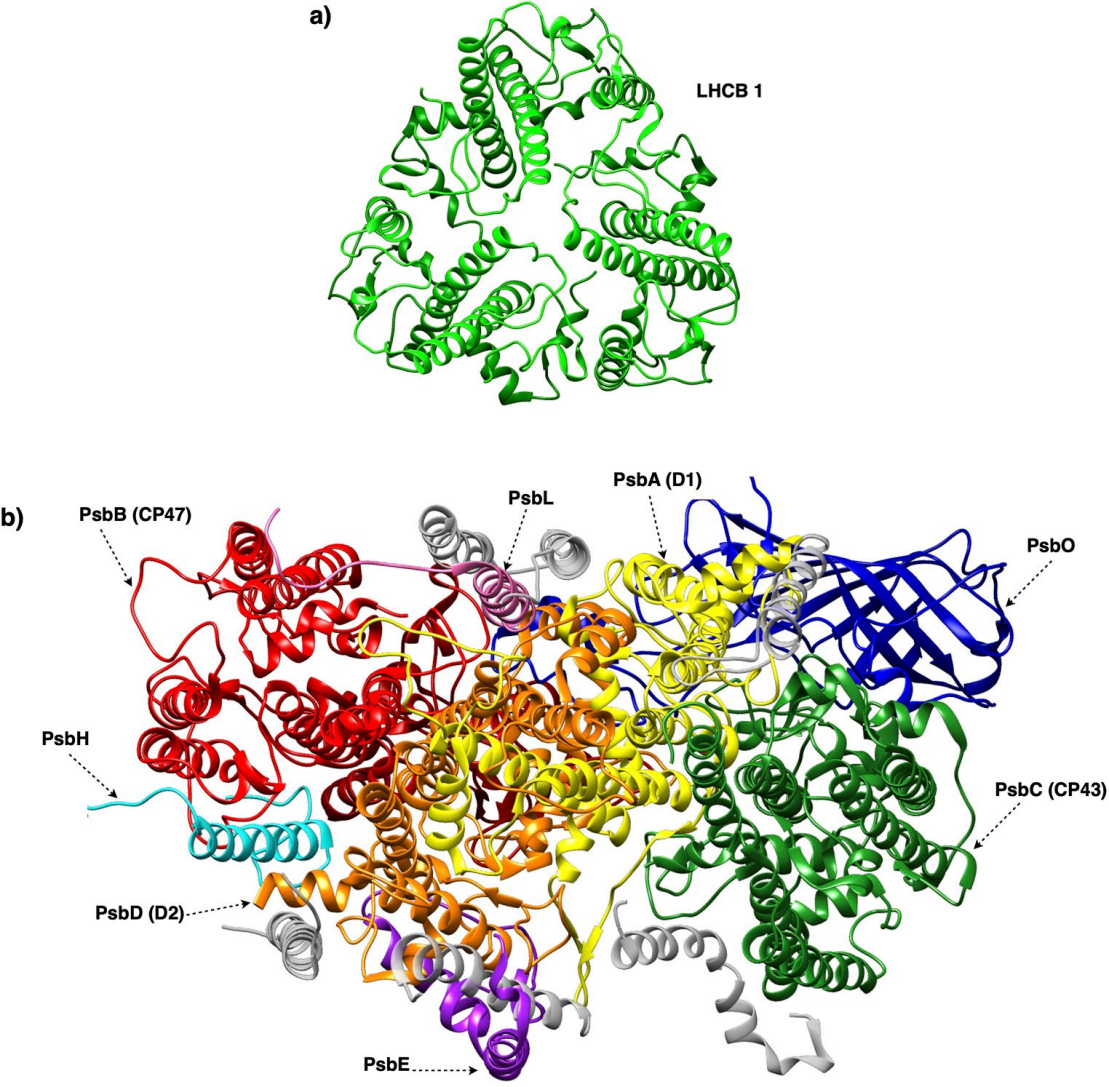
Ribbon representation of a LHCII trimer (**a**) and a PSII monomer (**b**) viewed from the stromal side. In both cases, the subunits identified by mass spectrometry are shown in color and identified by their name, whereas subunits not detected by mass spectrometry analysis are shown in grey. The LHCII and PSII models used in this figure is deposited in the RCSB with PDB codes 8Y15 and 1S5L, respectively

Bands A1 and B1 shared six different PSII subunits (PsbA, PsbB, PsbD, PsbE, PsbO, and PsbL) consistent with their assignment of PSII monomers. PsbC was identified only in band A1, whereas PsbH only in band B1; however, the PsbH entry in B1 was just below the threshold used to rank the relative representativity of the MS entries (Table S1). Bands A2 and B2 both shared a dominant Lhcb1 entry (CB22_MAIZE, Lhcb1.5), supporting their attribution to LHCII trimers. In addition, band B2, also contained CP24 (CB4A_SOLLC, Lhcb6), which is consistent with its possible association with a PSII pool. A graphical summary on the subunits identified for LHCII trimers (bands A2/B2) and PSII monomers (bands A1/B1) is presented in Fig. 6. At present, it remains unclear whether this monomeric state reflects a physiological configuration in *P. oceanica* or is a consequence of the solubilization procedure; further work employing milder extraction conditions is in progress aiming at clarifying this aspect.

Mass spectrometry clearly distinguished the molecular identity of the two chromatographic pools, revealing that the first solubilization predominantly contained chlorophyll *a–b* binding proteins (LHCII), whereas the second was enriched in PSII core subunits (Fig. 6; Tables 2 and 3). Beyond providing evidence that the isolation workflow yields structurally intact complexes, the MS-based subunit identification contributed to expanding and refining the proteomic resources available for this key marine angiosperm, an outcome that is particularly valuable given the current scarcity of molecular information for this species (Table 3). Moreover, the spectral properties of the two samples strongly supported the BN-PAGE/MS-based assignment (Fig. 5). The LHCII sample from the chromatography of the first solubilization pool showed the characteristic chlorophyll *b* and carotenoid-associated features (653 nm and 470 nm), while the PSII sample from the chromatography of the second solubilization pool exhibited the pheophytin and/or cytochrome b559 heme known to be related with the absorption peak at 415 nm (Nanba and Satoh 1987; van Amerongen and van Grondelle 2001; Son et al. 2020; Knoppová et al. 2022; Li et al. 2023).

Taken together, our results establish a methodology for the preparative isolation of thylakoids and their constituent complexes, enabling structural and functional studies of photosynthesis in *P. oceanica*, a species that has remained largely inaccessible to biochemical investigation despite its ecological importance. By overcoming the biochemical barrier imposed by polyphenols, and by demonstrating that intact photosynthetic complexes can be isolated by preparative chromatography, spectroscopically characterized, and confidently identified by mass spectrometry, this work provides a foundation for future analyses of the organization, regulation, and photophysiology of the photosynthetic apparatus of this marine phanerogam. Importantly, the proteomic identifications obtained here expand the currently limited sequence coverage available for *P. oceanica*, contributing new high-confidence entries that will facilitate future comparative, quantitative, structural, and evolutionary studies.

## Supplementary Information

Below is the link to the electronic supplementary material.

Supplementary Material 1

Supplementary Material 2

## Data Availability

No datasets were generated or analysed during the current study.
