# Supplementary material for: Preparative isolation and preliminary characterization of LHCII trimers and PSII monomers from Posidonia oceanica L
Source: Photosynth Res. 2026 Jan 29;164(1):10. doi: 10.1007/s11120-026-01196-3 (PMC12855319; doi:10.1007/s11120-026-01196-3)

6M SDS-PAGE used for Figure 3a.
Arrows indicate the lanes used in the figure.

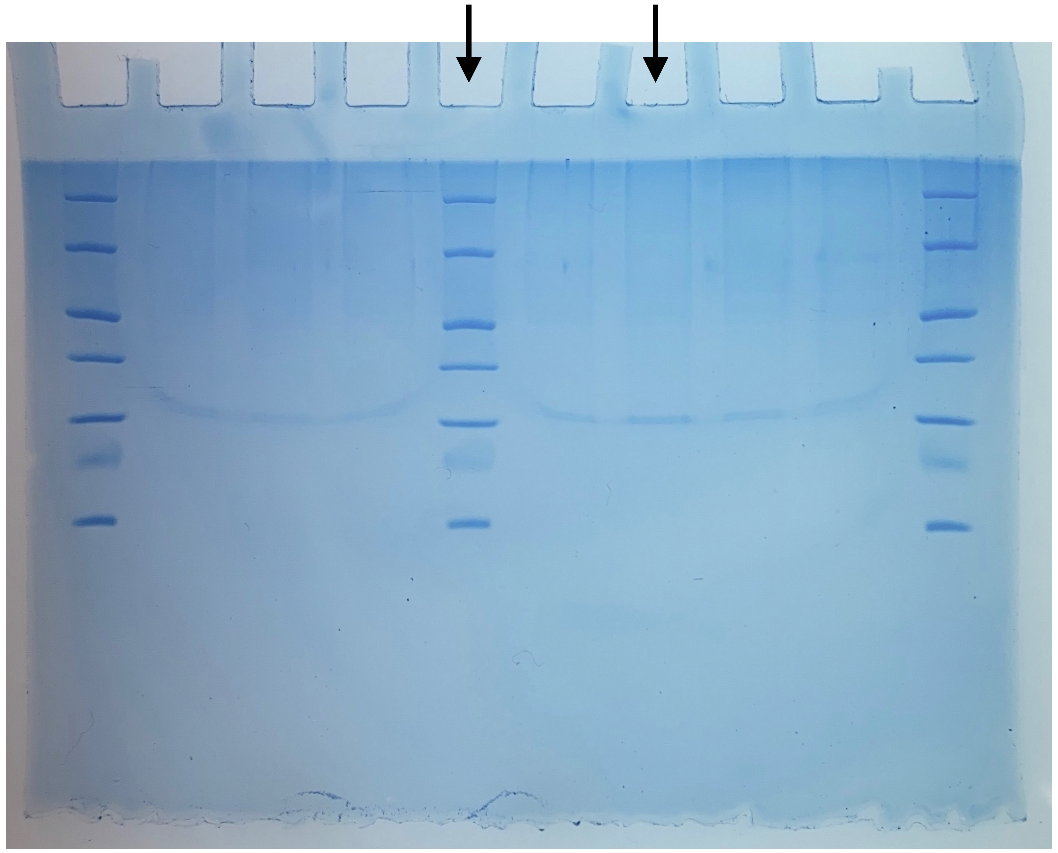

6M LiDS-PAGE used for Figure 3b.
Arrows indicate the lanes used in the figure.

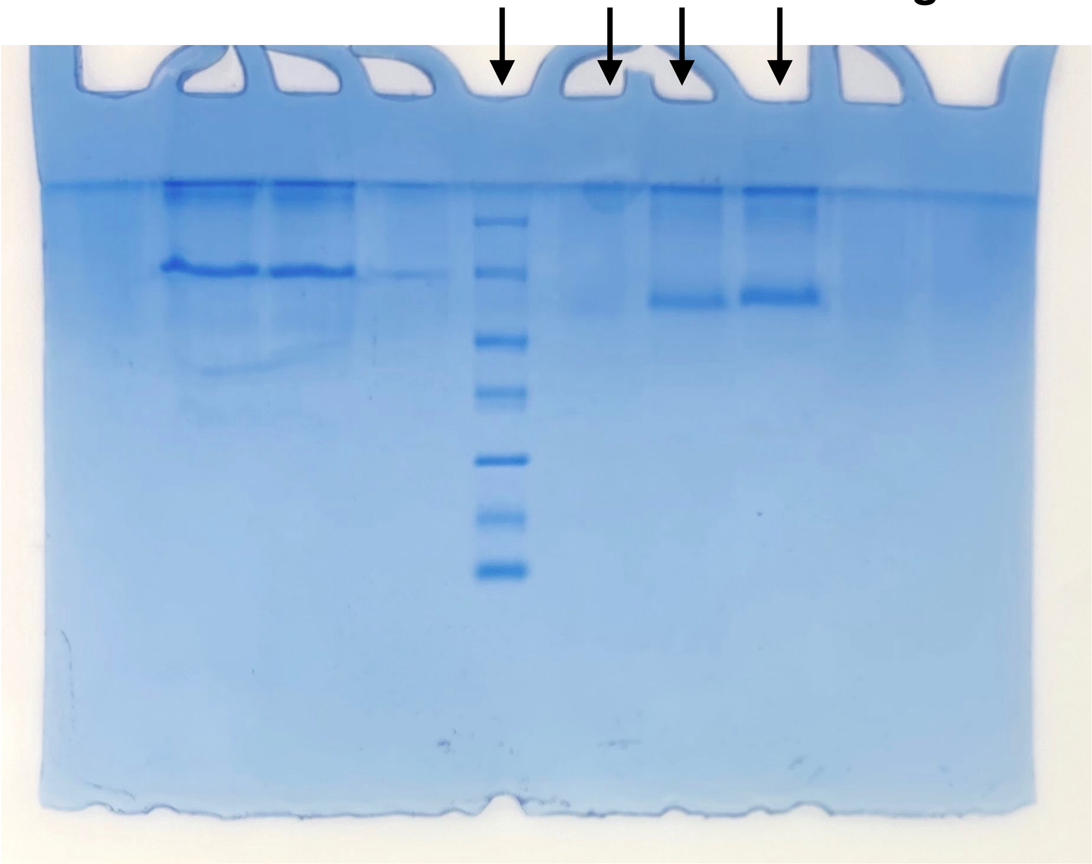

8M LiDS-PAGE used for Figure 3c.
Arrows indicate the lanes used in the figure.

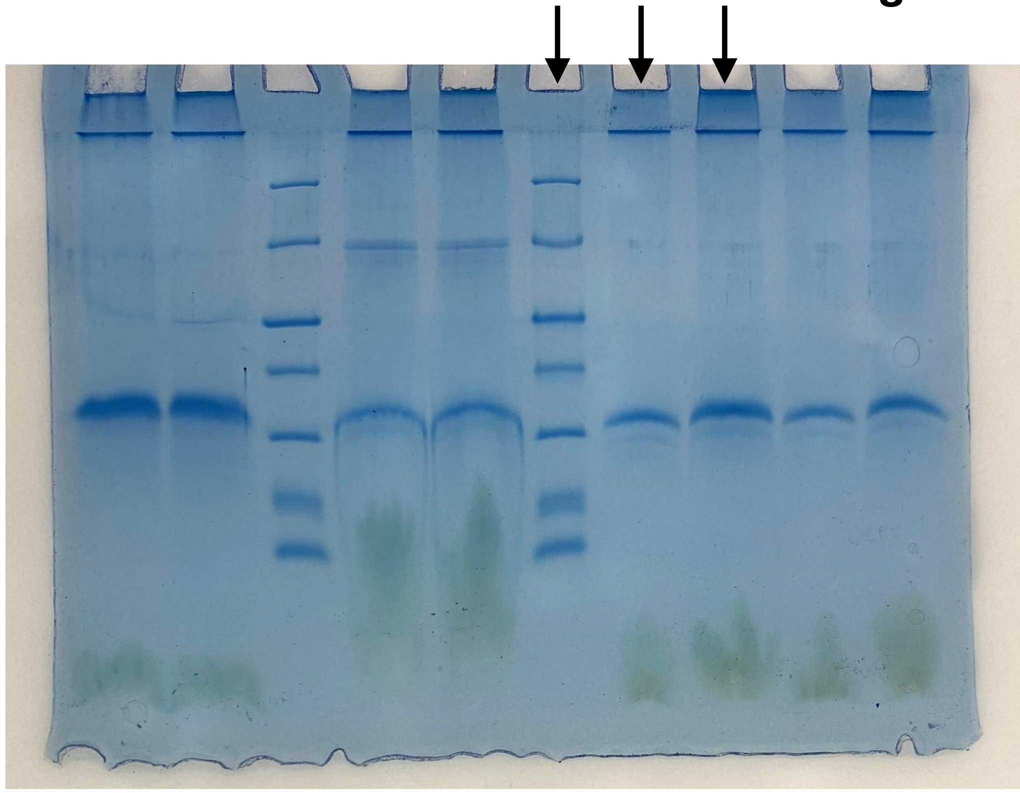

BN-PAGE used for insets in Figure 4.
Arrows indicate the lanes used in the figure.

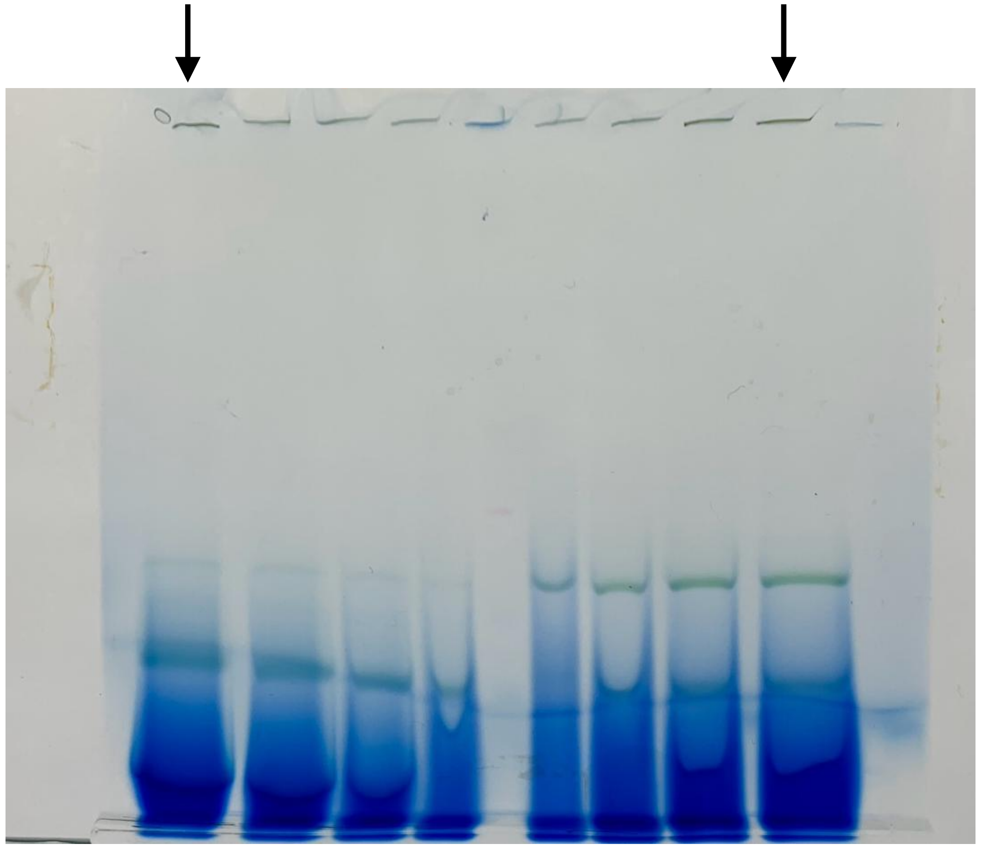

8M LiDS-PAGE used for Figure S1.
Arrows indicate the lanes used in the figure.

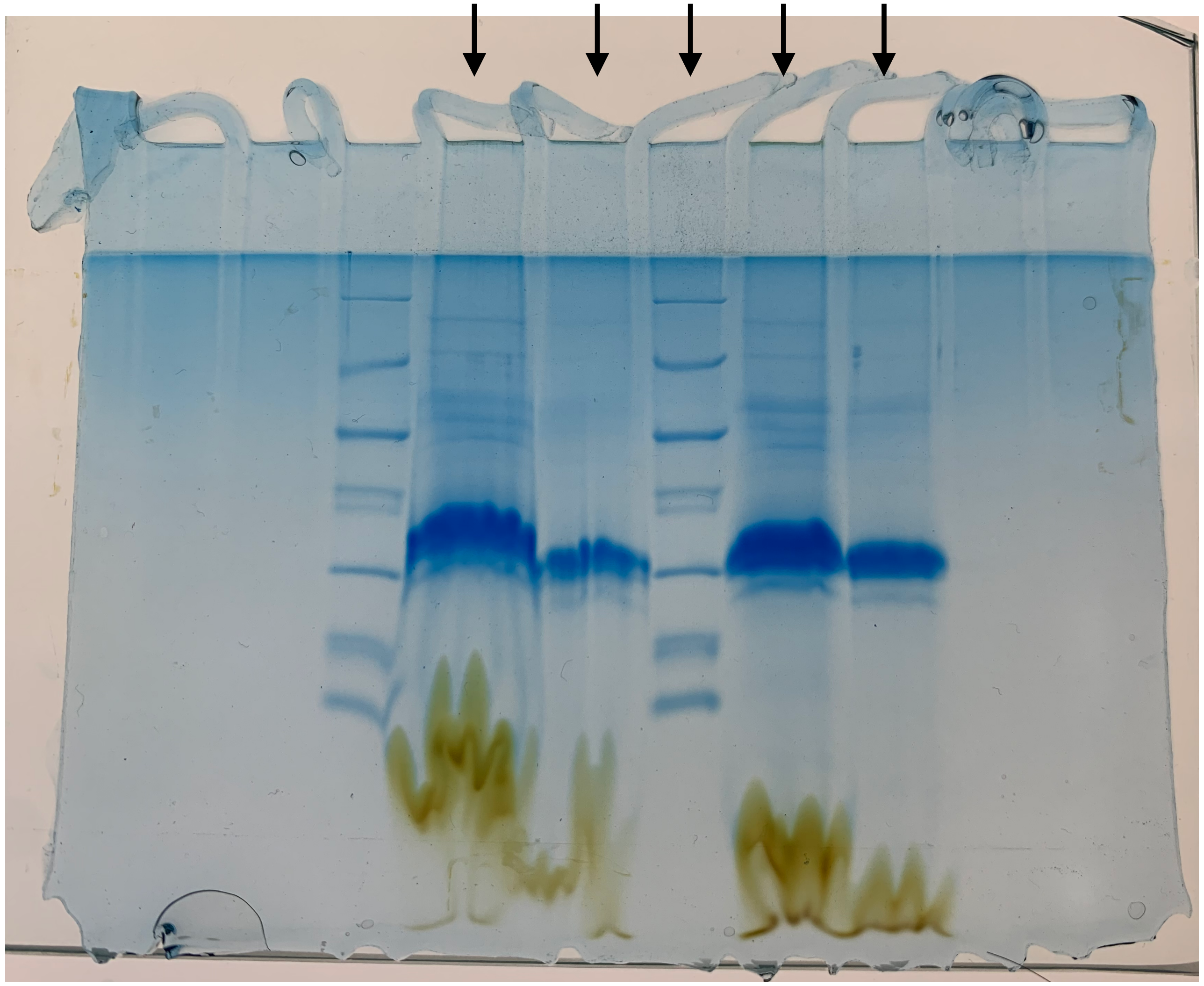

Supplement: Supplementary file 1 — Supplementary Material 1 [file 11120_2026_1196_MOESM1_ESM.pdf]
