# Supplementary material for: Preparative isolation and preliminary characterization of LHCII trimers and PSII monomers from Posidonia oceanica L
Source: Photosynth Res. 2026 Jan 29;164(1):10. doi: 10.1007/s11120-026-01196-3 (PMC12855319; doi:10.1007/s11120-026-01196-3)
Supplement: Supplementary file 2 — Supplementary Material 2 [file 11120_2026_1196_MOESM2_ESM.pdf]

Supplementary information for

Preparative isolation and preliminary characterization of LHCII trimers and PSII monomers from *Posidonia oceanica L.*

Stefano Francesco Farci[1], Luca Iesu[2], Domenica Farci[3*], Dario Piano[1*]

Affiliations

[1]Department of Life and Environmental Sciences, University of Cagliari, Cagliari, Italy.; [2]Université Paris-Saclay, Univ Evry, CY Cergy Paris Université, CNRS, LAMBE, 95000, Cergy, France; [3]Department of Plant Physiology, Warsaw University of Life Sciences SGGW, Warsaw, Poland.

**Corresponding authors: Prof. Dr. D. Piano (dario.piano@unica.it), Dr. D. Farci (domenica_farci@sggw.edu.pl).*

Content:

Supplementary Figure 1 (Fig. S1)

Supplementary Figure 2 (Fig. S2)

Supplementary Table 1 (Table S1)

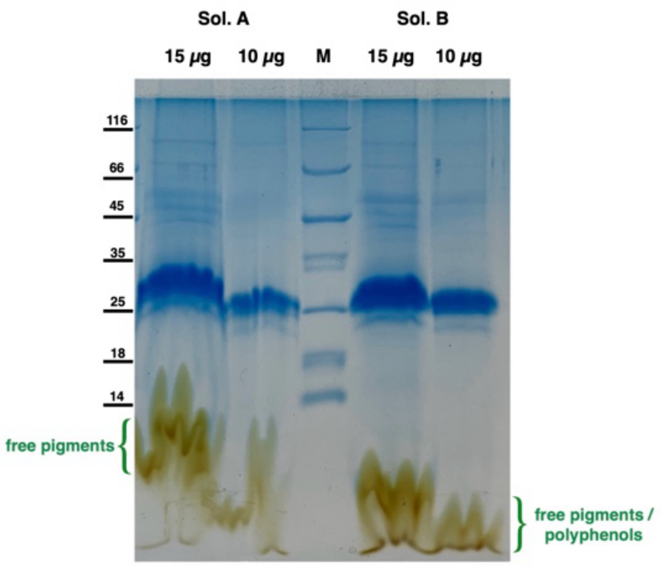

Fig. S1: Denaturing PAGE performed under optimized conditions (8 M urea and 10% LiDS) on pools derived from both solubilizations A and B. The total amount of protein loaded, expressed in µg, is indicated in the figure. M denotes the molecular weight marker. Free pigments and residual polyphenols in the migration front are indicated.

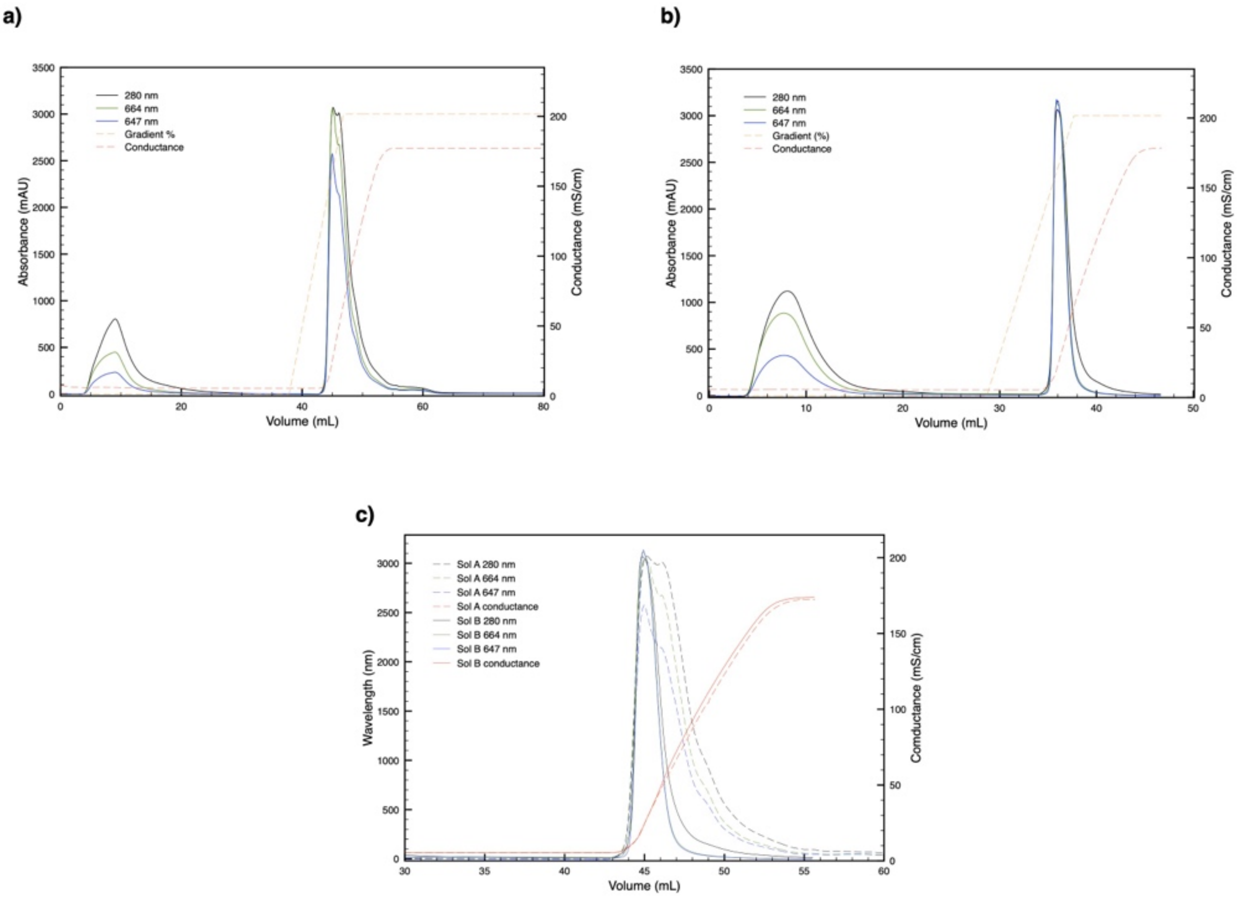

Fig. S2: Chromatograms including the applied gradients and the absorbance profile at 647 nm. The remaining profiles correspond to those shown in Figure 4. For further details, please refer to the Figure 4 caption.

Table S1: Entries, related accession numbers, and main MS data. For each entry, the accession number, entry description, and molecular mass are provided. Main MS data include the Mascot Score, number of matching spectra, number of matched sequences, and sequence coverage. For each entry, the MASCOT score normalized per mass unit and the corresponding index (as described in Table 1) are also reported. The first entry in A1 and B1, LHCII peptide (highlighted with a light blue background), is likely the result of partial co-migration, nonspecific interactions, and, based on the behavior of these samples during electrophoretic separation, aggregation or precipitation, consistent with the high abundance of LHCII in thylakoids. Entries with indices below 0.4 were considered less representative (highlighted with a light red background); this cutoff reflects relative abundance after correcting their score relatively to the protein size, and is based on the reference sample A1, in which all major PSII subunits have indices above 0.4.

BN-PAGE band	Accession No.	MASCOT score	MW	MW kDa	No. of matching spectra	No. of matching significant spectra	No. of matching sequences	No. of matching significant sequences	sequence coverage [%]	MASCOT / MW kDa	Quantitative presence (weighted)
A1	CB27_TOBAC	1942	28317	28.317	120	81	8	5	45.3	68.581	2.273
	PSBE_AMBTC	283	9381	9.381	10	10	3	3	37.3	30.167	1.000
	PSBA_ACOCL	928	38899	38.899	29	23	6	5	20.7	23.857	0.791
	PSBL_ACOCL	105	4494	4.494	2	2	1	1	36.8	23.364	0.775
	PSBD_ACOCI	866	39566	39.566	30	24	7	7	25.2	21.887	0.726
	PSBB_OENAR	1136	55962	55.962	40	26	10	7	23.2	20.299	0.673
	PSBO_PINST	180	12269	12.269	6	5	2	2	24.1	14.671	0.486
	PSBC_PHAAO	757	51756	51.756	31	22	12	9	30.2	14.626	0.485
	CYB6_AGRST	264	24151	24.151	13	11	4	4	19.1	10.931	0.362
	CB5_ARATH	310	30138	30.138	14	10	5	2	16.1	10.286	0.341
	CB121_HORVU	231	26447	26.447	12	8	3	2	8.2	8.734	0.290
	CB4A_SOLLC	220	27236	27.236	15	8	6	4	30.9	8.078	0.268
	CYF_HELAN	229	35182	35.182	14	11	5	5	17.2	6.509	0.216
	CB3_ARATH	104	28688	28.688	14	3	4	1	14.3	3.625	0.120
	CB2_MALDO	97	29193	29.193	4	3	3	2	9.3	3.323	0.110
	CA4_ARATH	82	27716	27.716	6	2	2	1	8.8	2.959	0.098
BN-PAGE band	Accession No.	MASCOT score	MW	MW kDa	No. of matching spectra	No. of matching significant spectra	No. of matching sequences	No. of matching significant sequences	sequence coverage [%]	MASCOT / MW kDa	Quantitative presence (weighted)
B1	CB22_MAIZE	4639	27977	27.977	275	209	6	6	26	165.815	5.536
	PSBE_AMBTC	281	9381	9.381	11	10	3	3	37.3	29.954	1.000
	PSBD_ACOCI	794	39566	39.566	27	22	7	5	26.9	20.068	0.670
	PSBB_OENAR	944	55962	55.962	31	20	10	7	26.2	16.869	0.563
	PSBO_PINST	203	12269	12.269	6	6	3	3	38.8	16.546	0.552
	PSBL_ACOCL	68	4494	4.494	1	1	1	1	36.8	15.131	0.505
	PSBA_ACOCL	548	38899	38.899	17	15	5	5	18.7	14.088	0.470
	CB121_HORVU	348	26447	26.447	16	8	3	2	8.2	13.158	0.439
	PSBH_PIPCE	91	7750	7.75	7	4	1	1	21.9	11.742	0.392
	CB2_MALDO	311	29193	29.193	18	11	4	2	10.1	10.653	0.356
	CYF_DIOEL	374	35248	35.248	26	12	7	4	18.8	10.611	0.354
	CYF_HELAN	373	35182	35.182	23	15	5	5	17.2	10.602	0.354
	CYB6_AGRST	231	24151	24.151	11	10	4	4	19.1	9.565	0.319
	CB23_SOLLC	242	28604	28.604	45	9	8	3	26.4	8.460	0.282
	PSBC_CYCTA	432	51795	51.795	21	12	8	7	23	8.341	0.278

BN-PAGE band	Accession No.	MASCOT score	MW	MW kDa	No. of matching spectra	No. of matching significant spectra	No. of matching sequences	No. of matching significant sequences	sequence coverage [%]	MASCOT / MW kDa	Quantitative presence (weighted)
	**UCRIA_SOLTU**	196	24251	24.251	4	4	3	3	16.1	8.082	0.270
	**PSBC_CUCSA**	399	51849	51.849	22	13	9	8	26.8	7.695	0.257
	**CB5_ARATH**	140	30138	30.138	10	4	5	2	16.1	4.645	0.155
	**CA4_ARATH**	93	27716	27.716	7	3	2	1	8.8	3.355	0.112
**BN-PAGE band**	**Accession No.**	**MASCOT score**	**MW**	**MW kDa**	**No. of matching spectra**	**No. of matching significant spectra**	**No. of matching sequences**	**No. of matching significant sequences**	**sequence coverage [%]**	**MASCOT / MW kDa**	**Quantitative presence (weighted)**
**A2**	**CB22_MAIZE**	6465	27977	27.977	348	261	7	6	42.3	231.083	1.000
	**CB4A_SOLLC**	2133	27236	27.236	78	58	10	9	45.3	78.315	0.339
	**PSBE_AMBTC**	351	9381	9.381	14	13	3	3	37.3	37.416	0.162
	**PSBO_PINST**	443	12269	12.269	14	10	3	3	38.8	36.107	0.156
	**PSBL_ACOCL**	122	4494	4.494	3	3	1	1	36.8	27.147	0.117
	**CB121_HORVU**	662	26447	26.447	28	16	4	3	9.8	25.031	0.108
	**PSBD_ACOCI**	980	39566	39.566	31	24	6	6	25.2	24.769	0.107
	**PSBH_PIPCE**	159	7750	7.75	10	6	2	2	23.3	20.516	0.089
	**PSBC_PHAAO**	978	51756	51.756	42	34	13	13	34.2	18.896	0.082
	**PSBA_ACOCL**	686	38899	38.899	23	19	5	5	18.7	17.635	0.076
	**CB5_ARATH**	525	30138	30.138	20	13	6	3	19.6	17.420	0.075
	**UCRIA_SOLTU**	355	24251	24.251	8	7	3	2	16.1	14.639	0.063
	**CB23_SOLLC**	358	28604	28.604	39	13	9	4	40.8	12.516	0.054
	**CYB6_AGRST**	264	24151	24.151	13	10	5	3	26.5	10.931	0.047
	**PSBB_ATRBE**	423	55979	55.979	14	10	10	6	25.4	7.556	0.033
	**PSAD_HORVU**	136	21919	21.919	8	3	5	2	28.8	6.205	0.027
	**CA4_ARATH**	132	27716	27.716	6	3	2	1	8.8	4.763	0.021
	**P2SAF_ORYSJ**	104	45441	45.441	5	4	4	3	10.3	2.289	0.010
**BN-PAGE band**	**Accession No.**	**MASCOT score**	**MW**	**MW kDa**	**No. of matching spectra**	**No. of matching significant spectra**	**No. of matching sequences**	**No. of matching significant sequences**	**sequence coverage [%]**	**MASCOT / MW kDa**	**Quantitative presence (weighted)**
**B2**	**CB22_MAIZE**	5105	27977	27.977	279	225	6	6	26	182.471	1.000
	**CB4A_SOLLC**	2022	27236	27.236	72	53	11	8	45.7	74.240	0.407
	**PSBE_AMBTC**	276	9381	9.381	16	13	3	3	37.3	29.421	0.161
	**CB121_HORVU**	748	26447	26.447	29	17	4	3	9.8	28.283	0.155
	**PSBO_PINST**	298	12269	12.269	8	6	3	3	38.8	24.289	0.133
	**PSBL_ACOCL**	106	4494	4.494	2	2	1	1	36.8	23.587	0.129
	**UCRIA_SOLTU**	554	24251	24.251	14	12	4	3	16.1	22.844	0.125
	**PSBD_ACOCI**	541	39566	39.566	14	11	5	5	20.4	13.673	0.075
	**CB5_ARATH**	388	30138	30.138	22	11	6	2	16.1	12.874	0.071
	**PSBA_ACOCL**	436	38899	38.899	16	12	6	5	20.7	11.209	0.061
	**CYB6_AGRST**	242	24151	24.151	13	7	5	3	26.5	10.020	0.055
	**PSAD_HORVU**	189	21919	21.919	8	6	4	3	21.5	8.623	0.047
	**CB23_SOLLC**	236	28604	28.604	36	13	8	3	26.4	8.251	0.045
	**PSBC_PHAAO**	410	51756	51.756	19	15	11	10	30.2	7.922	0.043
	**CA4_ARATH**	94	27716	27.716	6	2	2	1	8.8	3.392	0.019
	**TL17_ARATH**	81	25628	25.628	3	1	2	1	13.1	3.161	0.017
	**PSBB_ACOCI**	162	55962	55.962	5	4	3	2	6.9	2.895	0.016
